# Challenges in Complementing Data from Ground-Based Sensors with Satellite-Derived Products to Measure Ecological Changes in Relation to Climate—Lessons from Temperate Wetland-Upland Landscapes

**DOI:** 10.3390/s18030880

**Published:** 2018-03-16

**Authors:** Alisa L. Gallant, Walt Sadinski, Jesslyn F. Brown, Gabriel B. Senay, Mark F. Roth

**Affiliations:** 1 Earth Resources Observation and Science Center, US Geological Survey, 47914 252nd Street, Sioux Falls, SD 57198, USA; jfbrown@usgs.gov (J.F.B.); senay@usgs.gov (G.B.S.); 2 Upper Midwest Environmental Sciences Center, US Geological Survey, 2630 Fanta Reed Road, La Crosse, WI 54603, USA; wsadinski@usgs.gov (W.S.); mroth@usgs.gov (M.F.R.)

**Keywords:** wetland landscapes, water, evapotranspiration, snow-off, Normalized Difference Vegetation Index (NDVI), climate, start-of-season, growing season, scale, Moderate Resolution Imaging Spectroradiometer (MODIS)

## Abstract

Assessing climate-related ecological changes across spatiotemporal scales meaningful to resource managers is challenging because no one method reliably produces essential data at both fine and broad scales. We recently confronted such challenges while integrating data from ground- and satellite-based sensors for an assessment of four wetland-rich study areas in the U.S. Midwest. We examined relations between temperature and precipitation and a set of variables measured on the ground at individual wetlands and another set measured via satellite sensors within surrounding 4 km^2^ landscape blocks. At the block scale, we used evapotranspiration and vegetation greenness as remotely sensed proxies for water availability and to estimate seasonal photosynthetic activity. We used sensors on the ground to coincidentally measure surface-water availability and amphibian calling activity at individual wetlands within blocks. Responses of landscape blocks generally paralleled changes in conditions measured on the ground, but the latter were more dynamic, and changes in ecological conditions on the ground that were critical for biota were not always apparent in measurements of related parameters in blocks. Here, we evaluate the effectiveness of decisions and assumptions we made in applying the remotely sensed data for the assessment and the value of integrating observations across scales, sensors, and disciplines.

## 1. Introduction

Multidisciplinary research integrated across scales can provide resource managers and other stakeholders valuable, useful information they need to manage resources adaptively in the face of global change. Using remotely sensed data in concert with data measured on the ground to describe changes in ecological conditions and processes across landscapes is one such research approach. This approach potentially can be powerful, but poses nontrivial challenges due to an unavoidable mix of direct and proxy measurements taken at different scales [1]. Ground-based measurements are critical to understanding specific habitat changes and effects on populations, for example, but can be labor- and travel-intensive and limited in scale/replicate field sites and in their area of inference. Satellite-based observations can cover broader portions of the landscape unhindered by such limitations or physical boundaries and provide much-needed landscape context for ground measurements taken at finer scales. However, relating remotely sensed, broad-scale data to ecological events and processes measured at finer scales can be challenging [2,3]. Thus, selecting effective remotely sensed measurements that also are useful for linking information across scales requires careful consideration and evaluation.

We have been studying how seasonal weather patterns and climate affect environmental conditions and processes in temperate, wetland-rich landscapes in the U.S. Midwest. We study amphibian populations because individuals of most amphibian species require both wetland and upland habitats over the course of a year. Amphibians have semipermeable skin [4] and are ectothermic, which renders them sensitive to changes in water and temperature conditions across a landscape [4] and can make them useful bellwethers of changing environmental conditions [5]. Declines of amphibian populations reported from around the world provide evidence of their susceptibility to loss or alteration of habitat, climate change, disease and pathogens, exposure to harmful chemicals, commercial trade, and introduced predators, among other global-change factors (e.g., [6,7,8,9,10,11,12]).

Assessing the presence of water over time is important not only to understand vegetation responses and the statuses of amphibian populations on these landscapes, but also to understand the potential for effects on other sympatric species and on the values of vital water-dependent ecosystem services [13,14] these landscapes provide, such as floodwater and sediment storage, filtering of contaminants, recreation, and climate regulation [15,16]. The presence of water on a landscape can be highly dynamic [17,18] and affected directly by changes in climate [19,20,21]. But, the science community largely lacks comprehensive information on long-term relations between climate, water availability in palustrine wetlands, and the presence of water across linked upland areas and how amphibian populations respond to changes in these landscape conditions that are fundamentally essential to their persistence. Thus, wetland-upland landscapes generally are critically important to humans and biodiversity and are not well-characterized in terms of impacts from changing climate dynamics. These landscapes also are potentially well-suited for integrated studies based upon collecting data on the ground and via satellite sensors to help address the need for such fundamental information.

Since 2008, we have repeatedly measured wetland water levels and the presence of calling amphibians at select replicate wetlands within four study areas in the U.S. Midwest. Individuals of most amphibian species in these areas require wetlands for reproduction and adjacent upland habitats for foraging and, typically, overwintering. We annually deployed sensors to measure water levels and acoustic recorders to sample amphibian calls hourly throughout the growing season. We analyzed these data in concert with data from nearby automated weather stations to assess relations among amphibian breeding behavior and potential reproduction success, wetland surface-water availability, air temperature, and precipitation. Understanding how these interactions fit within the larger landscape requires data from a range of scales [22]. We incorporated remotely sensed data on snow presence, evapotranspiration, and vegetation phenology and condition in local landscape blocks to provide broader ecological context to what we learned at individual wetland sites. 

We used evapotranspiration (ET) and vegetation response as proxies for water availability in the landscape. ET represents the movement of moisture from waterbodies, soil, and vegetation to the atmosphere as a function of energy input, atmospheric demand, and aerodynamic conditions. ET therefore may afford us a more functional understanding of environmental conditions relative to moisture. Vegetation condition is informative not only about moisture availability, but also about the suitability of ambient air temperatures for plant growth. We can study annual differences in growing-season conditions by monitoring the timing and strength of the photosynthetic response, which we did using the Normalized Difference Vegetation Index (NDVI), a metric with a long history of use in the remote sensing community that has been applied throughout the world to map response of green biomass in visible-red and near-infrared wavebands (e.g., [23,24,25,26]). NDVI also has been linked with biodiversity in studies of trophic interactions [3]. We brought all these measurements into an integrated analysis of inter- and intraseasonal processes and characteristics across the study areas for the first five years of data collection, from 2008 to 2012 [27].

In analyzing these first five years of data, we considered the decisions and assumptions we made in selecting remotely sensed variables to use and determining how to summarize the data most effectively. Our results illustrate not only how differentially sensitive data from ground- and satellite-based sensors can be for detecting weather-induced, ecologically important changes in water availability, but also how nuanced the effects on results may (or may not) be from the decisions we made in applying the remotely sensed data in our analyses. In this paper, we evaluate the overall effectiveness of our approach in executing the assessment to determine if further refinements are needed before moving forward. We specifically consider whether: (1) the ET and NDVI variables that we used were sensitive to the onset of the growing season and to changes in growing conditions (moisture availability and air temperature) throughout the season in temperate, wetland-rich landscapes; (2) our units of analysis preserved the information from the variety of original resolutions of source datasets; and (3) the definitions we developed and implemented to capture events at the start of the growing season (snow-off, start of vegetation green-up, and onset of ET activity) ultimately were appropriate. Our findings potentially are relevant for other researchers intending to integrate ground- and satellite-based measurements for their studies.

## 2. Materials and Methods

### 2.1. Study Areas

**Figure 1:**
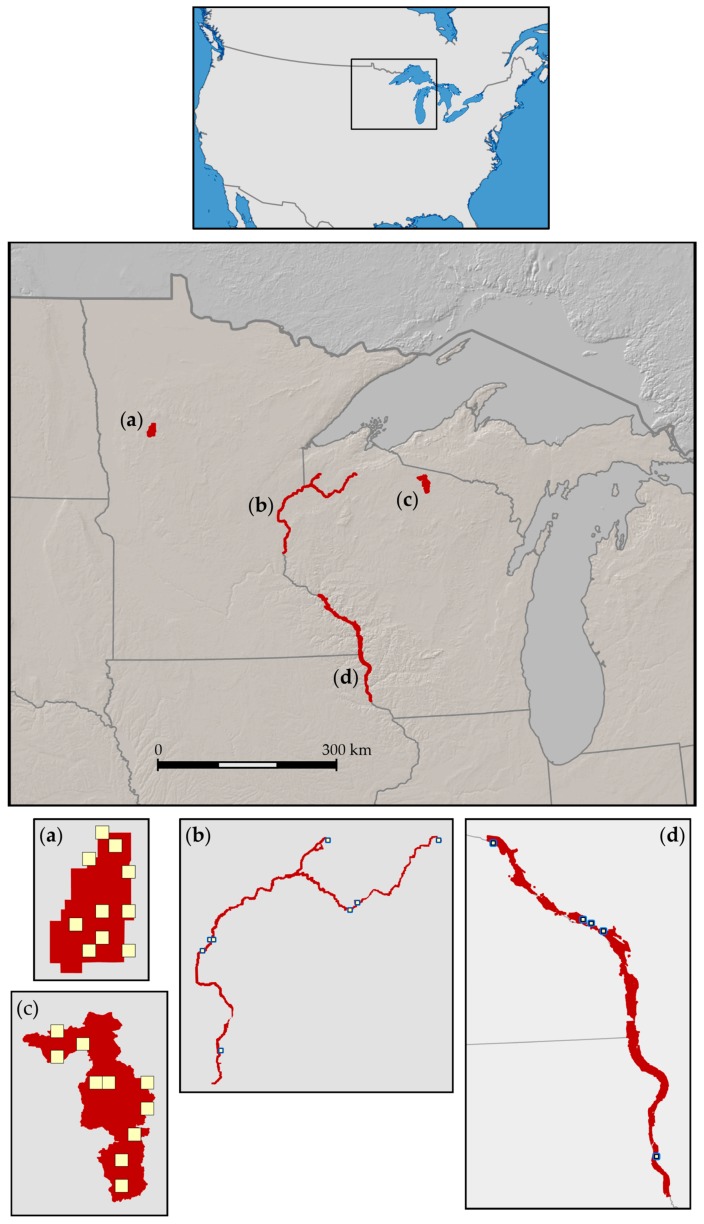
Map of the four study areas (in red): (**a**) Tamarac National Wildlife Refuge; (**b**) St. Croix National Scenic Riverway; (**c**) North Temperate Lakes Long-term Research Area; and (**d**) Upper Mississippi River floodplain. Inset maps show distribution of landscape blocks.

We studied palustrine wetlands and their surrounding, interconnected uplands in four areas in the U.S. Midwest (Figure 1): Tamarac National Wildlife Refuge (“Tam”, inference area 182 km^2^) in Minnesota, St. Croix National Scenic Riverway (“SC”, inference area 331 km^2^) in Wisconsin and Minnesota, North Temperate Lakes Long-term Research Area (“NTL”, inference area 229 km^2^) in Wisconsin, and Upper Mississippi River floodplain (“UMR”, inference area 621 km^2^) in Wisconsin. Tam is located in a transitional zone of tallgrass prairie, eastern broadleaf forest, and northern needleleaf forest and is dotted with lakes, temporary ponds, marshes, bogs, and swamps [28]. SC is a network of protected riverways vegetated with fine-scaled patches of broadleaf, needleleaf, and mixed forests, shrubland, and tallgrass prairie intermixed with wet meadows, marshes, swamps, bogs, and fens [29]. NTL sites are within the Northern Highland-American Legion State Forest, a managed timber resource with stands of needleleaf, broadleaf, and mixed forests rich in lakes, ponds, marshes, bogs, and swamps [30]. The UMR includes the Upper Mississippi River National Wildlife and Fish Refuge, the Trempealeau National Wildlife Refuge, and Perrot State Park and is characterized by floodplain marshes and meadows, bottomland hardwoods, grasses, and shrubland, and adjacent uplands of broadleaf forest [31,32]. UMR wetlands are on the list of Ramsar Convention wetlands of international importance [33]. 

We sampled individual wetlands on the ground to characterize how temperature and precipitation were associated with surface-water availability and amphibian calling activity (which is related to their breeding). We studied 2 km × 2 km landscape blocks that included our individual wetland sites with satellite-derived variables of moisture availability and vegetation response to compare how they related to ground-based temperature and precipitation and responses we measured at individual wetlands. 

### 2.2. In-Situ Measurements

We designed a satellite-based assessment approach that would complement our ground-based measurements at wetland sites. We briefly describe the in-situ measurements here because (1) they were the basis for determining the locations of the landscape blocks; (2) they provided support for our use of precipitation and air temperature data from weather stations outside the landscape blocks; and (3) we include example results from in-situ observations in connection with the remotely derived variables later in this paper.

Wetland sites were selected by positioning a rectangular grid of 25 ha cells across each study area, randomly selecting cells (10 from Tam, SC, and NTL and five from UMR), then surveying the selected cells from the ground to locate the first wetland in each cell that we confirmed was, or that appeared to be, an amphibian breeding site [27]. We deployed field sensors at individual wetlands beginning in 2008 (details provided in [27]). Acoustic recorders were installed as soon as thawing permitted and prior to when amphibians began calling to record amphibian calling hourly throughout each breeding season. We installed pressure sensors and measured water levels hourly throughout the reproduction season at all wetlands except those that were too deep to wade or were not accessible by kayak or canoe. In 2012 we deployed air-temperature loggers at all study wetlands to evaluate how closely temperature data we obtained from nearby weather stations aligned with conditions at individual sites. We similarly installed a tipping-bucket rain gauge at two wetland sites each for Tam, SC, and NTL to compare daily rainfall observations with rainfall recorded at nearby weather stations. From the rainfall and air temperature records we determined that weather conditions at our wetland study sites were similar to those measured at nearby weather stations. We therefore assumed the longer-term data record from weather stations (described below) provided reasonable representations of weather conditions at our sites for both ground- and satellite-based analyses.

### 2.3. Data from Weather Stations

We used daily data from the weather stations nearest to our field sites and associated landscape blocks that had the most complete set of annual records for 2008–2012 to relate with satellite-derived variables to determine the onset of growing conditions for plants and the influence of seasonal precipitation and air temperature dynamics. We selected automated over analog weather stations when we had the choice. We selected 12 stations to provide data for the 33 landscape blocks that contained our 35 field sites (note, in two cases a single landscape block encompassed two wetland field sites; see Appendix A for descriptions of “primary” weather stations). We screened weather data for completeness and for spurious entries (e.g., excessively high air temperatures in winter months) and replaced spurious or missing observations with data from five additional, nearby stations when deemed appropriate (“mitigation” stations in Appendix A).

We analyzed temperature and precipitation data to evaluate how well satellite-derived variables for ET and NDVI related to the onset of growing-season temperatures and seasonal accumulated precipitation. We used growing degree units (“GDU”, also commonly called growing degree-days) as a metric that translated air temperature into units meaningful for plant growth and ET response. GDU represent the number of degrees the average daily temperature exceeds a baseline during a 24 h period and generally are cumulated over the growing season [34]. The concept typically is used to estimate seasonal conditions for timing and development of different crop types [35], and the baseline is selected accordingly [36,37]. Scientists have employed GDU successfully to interpret NDVI responses (e.g., [36,38,39,40,41]). 

We used 10 °C as a baseline temperature for assessing vegetation response (represented by NDVI) to air temperatures and set an upper limit of 30 °C, above which we expected growth rates to slow. This range commonly is applied for the major crop types grown in the areas we studied, although our specific research sites are non-agricultural and may have thermal limitations that differ from crops. We implemented the GDU calculation following Method 2 in McMaster and Wilhelm [35]. We examined relations between the onset of GDU, which we defined as the first week of the year that median daily GDU was ≥1, and the first week we observed the start-of-season production of green biomass (via NDVI) and a total ET ≥1 mm. We used a weekly time step to summarize the daily weather observations because our satellite-derived products (described below) were based on seven-day and eight-day composite intervals.

### 2.4. Satellite-Derived Measurements

We applied a weekly time step to standardize analysis units across ground- and satellite-based variables. 1–7 January represented week 1; 8–14 January represented week 2; and so on through week 34, around the end of August after the growing season had peaked (see Appendix B, Table A2, Table A3 and Table A4 for cross-referencing the various product time steps with weeks). We converted all satellite-derived products to a common map projection, the Albers equal-area conic projection typically used by the US Geological Survey for products spanning the conterminous United States (e.g., see metadata for [42]).

As described in Section 2.1, we analyzed satellite-based variables at the scale of landscape blocks to assess their temporal relations with ground-based measurements of GDU and precipitation [27]. We chose 2 km × 2 km as the block size because it accommodated the approximate averages of published home ranges and maximum distances traveled for amphibian species that live in our study areas [43,44,45,46]. This block size, which was larger than the pixel sizes of the remote-sensing products we used, also helped to overcome an inhibiting effect on NDVI and ET responses that could have occurred when standing water occupied a large proportion of a pixel in our study areas. Moreover, the NDVI product we used had masked areas of perennial open water [47], which sometimes included our study wetlands (blocks locations are available at [48]). We delineated landscape blocks by overlaying a 2 km × 2 km grid across the Midwest and extracting those cells that contained the study wetlands. In this paper, we consider how the decision to use landscape blocks might have influenced results compared with results we would have obtained analyzing data from an individual pixel of ET, NDVI, or Snow associated with an individual wetland study site.

#### 2.4.1. ET

We used a dataset of estimated *actual* (in contrast with estimated *potential*) ET that is being generated operationally for the conterminous United States [49]. The dataset is produced with the Operational Simplified Surface Energy Balance (SSEBop) model, which uses an energy-balance approach to estimate ET, applying remotely sensed land-surface temperature to solve for the latent heat energy component as a product of ET fraction (0–1.0) generated from land-surface temperature and atmospheric demand (potential ET) estimated using the Penman-Monteith equation. Output from the model has a spatial resolution of 1 km^2^ and represents total actual ET in millimeters per eight-day interval throughout the year [49]. Principal inputs to SSEBop are eight-day average land-surface temperature from the Moderate Resolution Imaging Spectroradiometer satellite sensor (MODIS; product MOD11A; see [50]), air temperature in the form of gridded surfaces interpolated and extrapolated from daily meteorological observations by the Daily Surface Weather and Climatological Summaries (DAYMET) model [51,52], and potential ET [53] derived from weather fields produced by the Global Data Assimilation System (GDAS; [54]). A comprehensive performance evaluation of the SSEBop model in the conterminous United States has been reported by Velpuri et al. [55].

We obtained estimated actual ET data in their native latitude/longitude system for our study areas for 2008 through 2012 (a similar global product is available online [56]). We fitted the data to the 4 km^2^ block framework by calculating the average estimated ET across the four pixels within each block (data available from [48]). We assigned each eight-day composite period of ET output to the week to which it best aligned (Table A4). The 46 eight-day composite intervals per year left us without matches for week numbers 5, 13, 21, and 29 from January through August, which we treated as missing data in our statistical analyses. We compared the onset of ET activity (i.e., first week of the year when total ET was ≥1 mm) with the timing of the onset of growing degree conditions. We also evaluated (correlation analysis) whether the ET values at the block scale differed much from those at the scale of the individual pixel associated with each wetland site.

#### 2.4.2. NDVI

We used an NDVI product generated with data from “eMODIS” (available online [57]), a system developed at the US Geological Survey’s Earth Resources Observation and Science (EROS) Center [47,58]. The EROS NDVI dataset was derived with input from Collection 5 level-1B files from both Terra and Aqua MODIS. Data represented atmospherically corrected surface reflectance at a spatial resolution of 250 m processed as seven-day rolling composites. These data were further processed to remove noise from snow, clouds, atmospheric haze, and other interference as part of the EROS phenology database [59]. This approach applied a weighted least-squares linear regression to a moving temporal window to calculate a regression line for each time step in the series [60]. The resulting family of regression lines associated with a data point was averaged for each point and interpolated between points to yield a continuous, smoothed vegetation signal. The smoothing process incorporated a weighting factor that favored peak points over sloping or valley points to lessen the influence of environmental or instrumental phenomena that introduce noise and typically reduce NDVI values. This approach provides a statistical basis for smoothing the data, and the smoothed NDVI curves provide a closer approximation of the growth cycle than do the original, noisy NDVI data. 

We obtained these smoothed NDVI data in their native Lambert azimuthal equal-area projection. We also obtained a derivative product identifying the annual start-of-season time (SOST), served through the EROS phenology database portal [59,61]. The SOST metric was produced via a curve derivative method that employed a backward-looking or delayed moving average [25,62]. Delayed moving average values were predicted based on previous observations along the time series NDVI curve for each pixel to identify departures from the established trend. The point (day of year) where the NDVI values first became larger than those predicted by the delayed moving average was labeled as the start-of-season time.

We converted the smoothed NDVI data to a weekly time step based on the week in which the majority of the (rolling seven) composited days occurred (refer to Table A3). We then fitted the NDVI data to the 4 km^2^ block framework by calculating the average NDVI value across all 250 m pixels within each block (data available from [48]). We also averaged the day-of-year timing identified by the SOST metric across the pixels within each block and subsequently checked the sensitivity of SOST through correlation with the onset of growing degree temperatures. We evaluated (correlation analysis) how NDVI values at the scale of individual pixels compared with values averaged across blocks. A characteristic of the original NDVI data products was that water pixels were masked (treated as “no data”) to eliminate spurious results for non-vegetated pixels. Some of our field study sites coincided with water-masked areas. In such cases we used the SOST from an adjacent, non-masked pixel (that we subjectively determined represented the wetland locale) to compare results based on wetland pixels with those based on study blocks.

Our initial analysis of the SOST metric revealed that its algorithm may, at times, have identified false green-up events during winter through early spring. False green-up can result from cycles of snowmelt followed by new snowfall, which exposes and then re-blankets evergreen and senescent vegetation. To help ensure we did not treat false green-ups as real, we derived an alternate start-of-season metric (altSOST). We visually examined the NDVI response curves from 2008 to 2012 for each landscape block to determine the winter background level for vegetation, which we defined as the highest NDVI values during the heart of winter. We calculated the average of the background levels across all blocks within each study area and used these averages (Tam = 0.34, SC = 0.45, NTL = 0.51, UMR = 0.31) as thresholds to signal when a rising NDVI curve had exceeded the winter background NDVI. We correlated the resulting altSOST dates with GDU to help evaluate whether the altSOST metric provided a more useful indicator than SOST for the onset of green-up.

#### 2.4.3. Snow-Off

We used a NASA-supported product generated from MODIS Collection 5 data (MOD10A2 [63]) and provided in a sinusoidal projection to characterize the timing and duration of snowpack. The product represented maximum snow extent for each eight-day composite period at a spatial resolution of 500 m. A pixel was labeled as “snow” if it had snow cover on one or more days during the composite period.

We assembled time-series snow data from 2008 to 2012 to obtain the snow status of the pixels within the block encompassing each study wetland. We determined the snow-free date for the pixels within each block and averaged the dates to derive a block-wide estimate (data available from [48]). We defined “snow-free” as the beginning date of two contiguous, snow-free, eight-day composite intervals that occurred after February (i.e., after the winter months). We imposed the requirement for two intervals because the U.S. Midwest often receives intermittent snowfall during late winter through spring. Snow from these late storms tends to be ephemeral, often melting within the same 24 h period in which it falls, as air temperatures become milder and photoperiods become longer. We ignored consecutive snow-free intervals when they occurred during winter months because plant growth was unlikely, given cold air temperatures (and assumed cold soil temperatures) and short photoperiods. We evaluated the suitability of our criteria for identifying the start of seasonal snow-free conditions by determining how often snow recurred after the snow-free date we identified. We also compared the differences of pixel-scale versus block-scale snow-off dates (correlation analysis) to assess any apparent bias based upon scale.

#### 2.4.4. Integrated Analyses

We brought together data from the ground and remote sensors to learn the extent to which they respectively tracked changes resulting from temporal patterns of precipitation and air temperatures at the wetland study sites/blocks. We focused our evaluations on: (1) ecological events around the onset of the growing season, a critical time for biota [64,65,66,67]; and (2) seasonal trajectories of responses past the peak of the season (near the end of August). In this paper, we share examples of results from the wetland ground-based data to highlight considerations and issues related to complementing these measurements with satellite-based measurements. Specifically, we examined the relative sensitivity of the remote measurements to changes in moisture availability and air temperature on the ground, versus the sensitivity to these changes exhibited by the in-situ measurements, and considered the ways in which the coarser spatial and temporal scales of the remote data could influence perceptions of conditions on the ground. 

## 3. Results

### 3.1. Start of Season

#### 3.1.1. Weather Characteristics, Including Snow Cover

**Figure 2:**
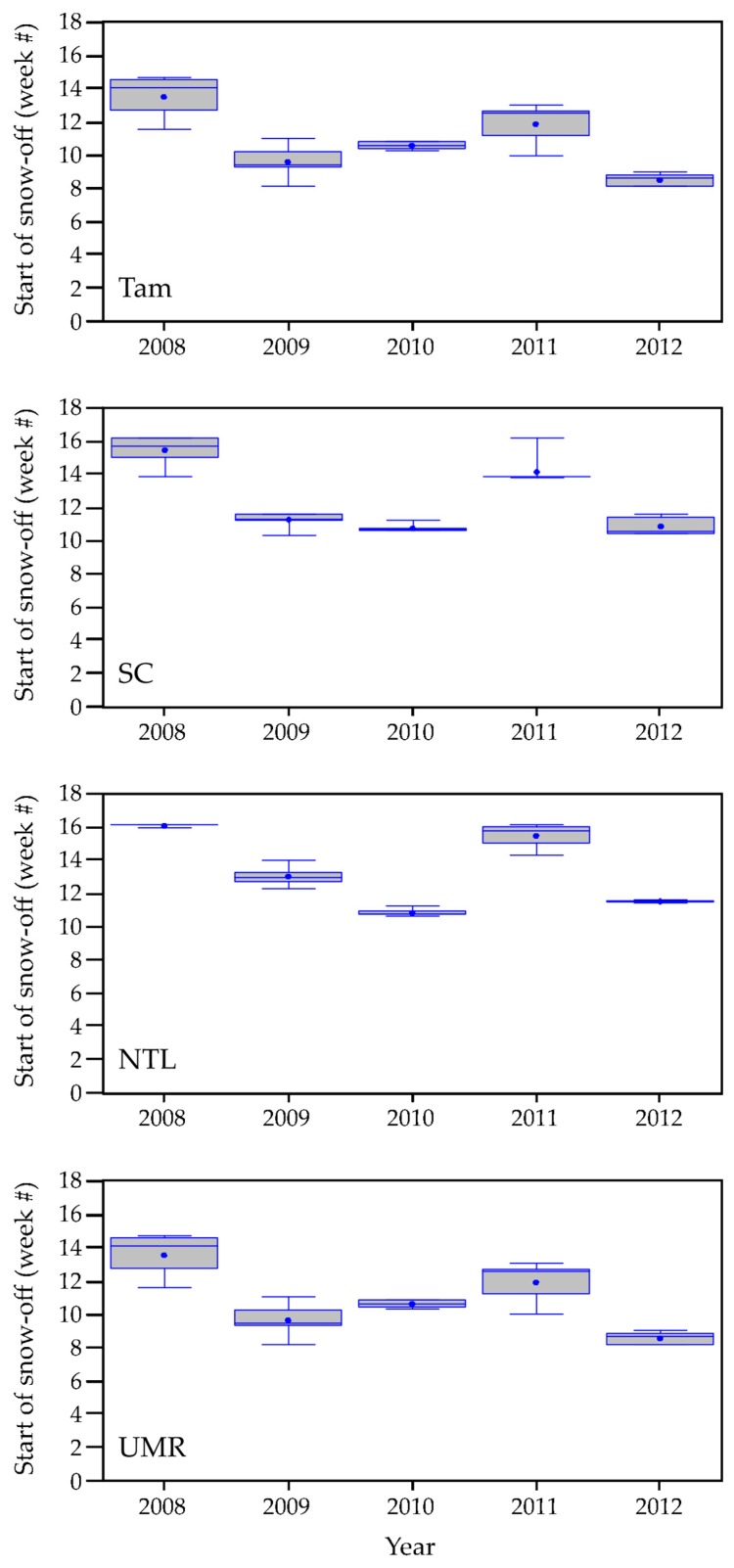
Timing of snow-off. These boxplots show average snow-off timing (beginning date of the first two consecutive, post-February snow-free intervals) across the set of 4 km^2^ blocks assessed within each study area. Tam = Tamarac National Wildlife Refuge. SC = St. Croix National Scenic Riverway. NTL = North Temperate Lakes Long-term Research Area. UMR = Upper Mississippi River floodplain.

**Figure 3:**
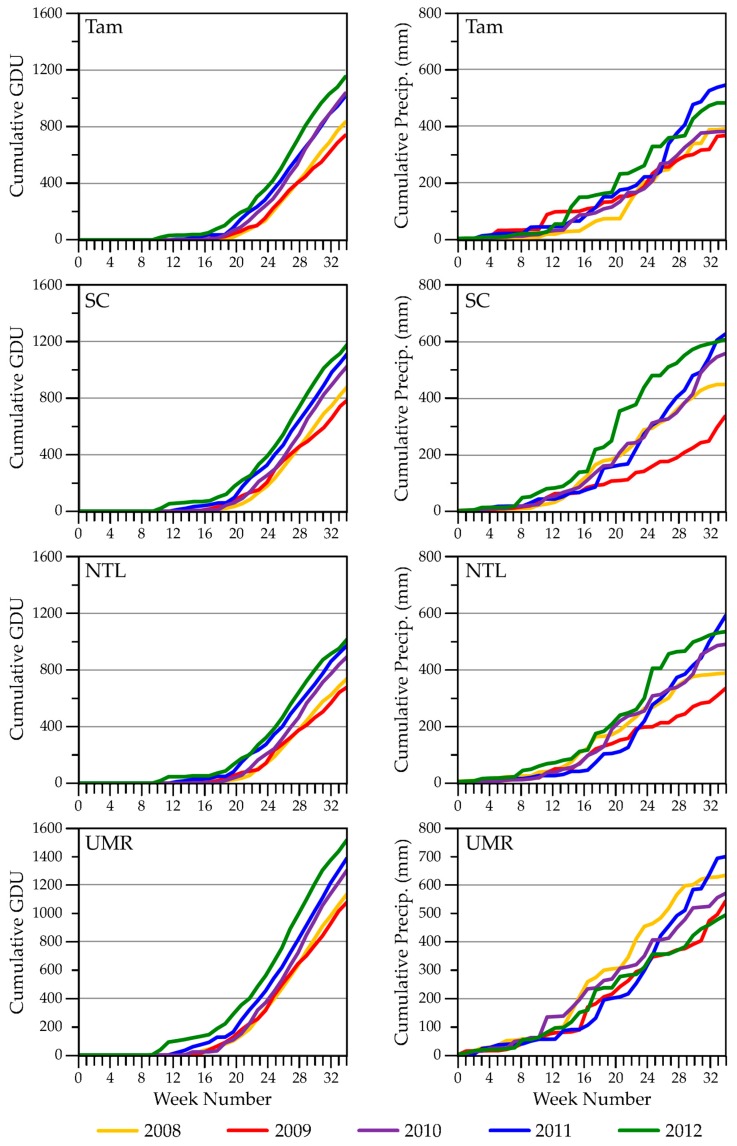
Cumulative growing degree units (GDU) and precipitation averaged across the weather stations we used for each study area (see Appendix A for list of stations). Tam = Tamarac National Wildlife Refuge. SC = St. Croix National Scenic Riverway. NTL = North Temperate Lakes Long-term Research Area. UMR = Upper Mississippi River floodplain.

Summarizing key weather characteristics helped us interpret how well our remotely derived metrics performed and highlighted patterns of seasonal variation. Years with earlier snowmelt, 2010 and 2012 at all our study areas (Figure 2), generally also were years when growing degree temperatures occurred earlier (Figure 3). Conversely, years with later snowmelt, 2008 and 2011 at all study areas, were years when low temperatures lingered later into the year. The year 2009 contrasted with these patterns by having early snowmelt at the SC, NTL, and UMR study areas, but accompanied by cooler air temperatures. The onset of growing degree temperatures occurred a month or more sooner in an early year than in a later year. Rainfall occurred earlier in both SC and NTL during 2012 in addition to the earlier warm air temperatures. In contrast, rain fell earlier at the Tam study area in 2009. Rainfall occurred very early in all years in the UMR.

Regarding our criteria to identify snow-free conditions, results indicated that, had we required only a single snow-free interval following February to designate snow-off, we would have observed recurrence of snow a substantial percent of the time at all study areas (Table 1, column (a)). By requiring two consecutive snow-free intervals we identified a snow-free date that rarely was followed by additional snowfall (Table 1, column (b)). We compared the timing of snow-off between the scales of individual 500 m pixels and study blocks associated with each wetland site and found them to be highly correlated (Table 1, column (c)). Values ranged from a Pearson’s correlation coefficient of 0.99 for SC to 0.93 for UMR.

#### 3.1.2. Onset of ET Activity

We observed some differences among years in total ET during spring months (Figure S1), but the ET data did not appear sensitive to the timing of the onset of growing degree temperatures. Interannual differences in spring monthly total ET were perhaps most distinctive in Tam, the study area with the most diversity in land-cover features (Figure S1). Interannual variability in spring ET activity was least evident in NTL blocks and UMR blocks.

**Figure 4:**
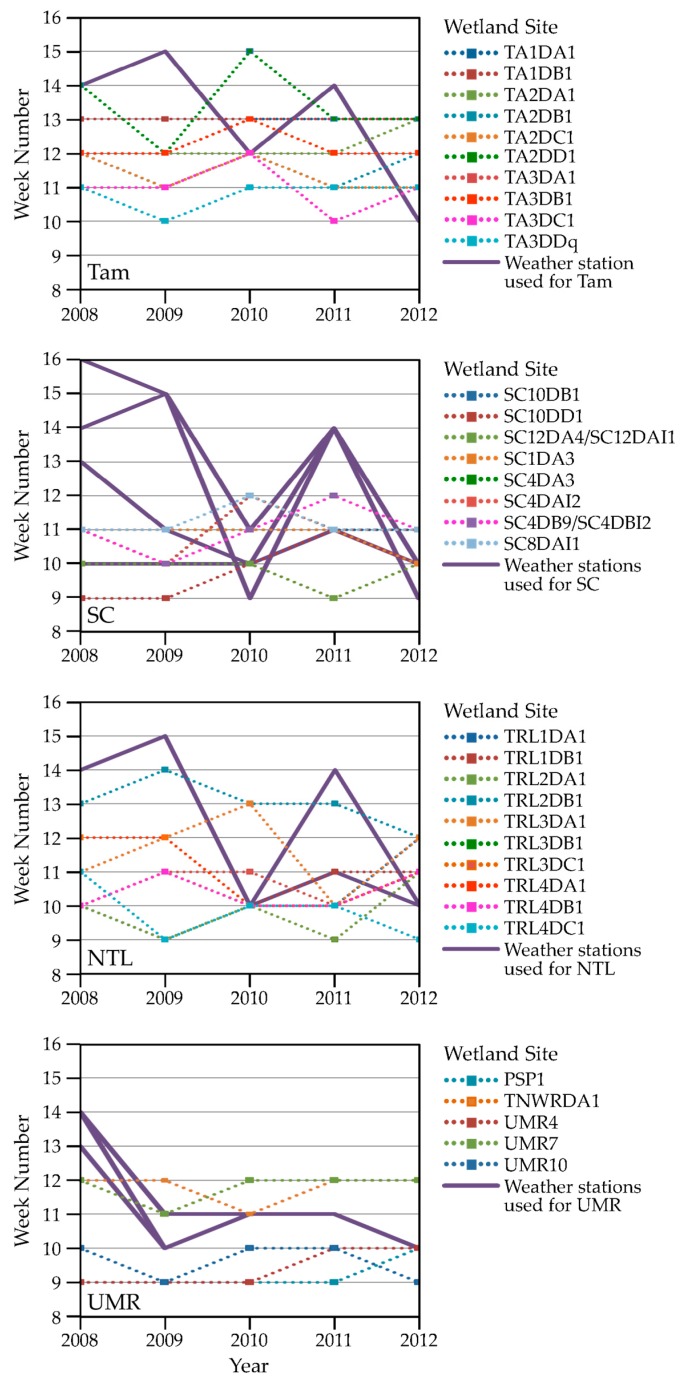
First week of the year when total weekly evapotranspiration was ≥1 mm for study blocks (symbols connected by dotted lines) compared with first week that median weekly growing degree units were ≥1 at weather stations associated with the study blocks (solid purple lines). Tam = Tamarac National Wildlife Refuge. SC = St. Croix National Scenic Riverway. NTL = North Temperate Lakes Long-term Research Area. UMR = Upper Mississippi River floodplain.

We used air temperature data from nearby weather stations to determine when the median weekly growing degree units first equaled or rose above 1 each year (solid purple lines in Figure 4). We compared these dates with the timing of when total weekly ET values first began to rise in study blocks (dotted lines in Figure 4). For a given landscape block, we found that the onset of ET activity was similar from year to year and did not mirror the interannual pattern of the onset of growing degree temperatures. For example, the onset of GDU for Tam was different each year, ranging from week 15 in 2009 to week 10 in 2012; yet, most blocks showed little variation in the annual initiation of ET activity. The NTL study area exhibited the greatest interannual variability in the onset of ET activity, but the temporal patterns bore little relation to the annual timing of the onset of GDU. 

Onset of ET activity was earlier than growing degree temperatures in 2008 in all blocks in all study areas, and in 2009 in all Tam blocks and some SC and UMR blocks, though not in NTL blocks (Figure 4). In 2011, the onset of ET activity preceded growing degree temperatures in all Tam blocks and in some blocks at the other three study areas. The onset of ET activity aligned best with the onset of growing degree temperatures in 2010 and 2012. Temperatures sufficiently above freezing to potentially cause evaporation (but too low for plants to respond, i.e., transpire) occurred by week 9 (the end of February) in all years for SC, NTL, and UMR, and in 2009, 2010, and 2012 for Tam (temperatures above freezing were first observed in week 10 for 2008 and 2011 at Tam). Hence, although the onset of ET activity preceded growing degree temperatures, air temperatures were above freezing when we identified initial ET activity.

#### 3.1.3. Vegetation Green-Up

**Figure 5:**
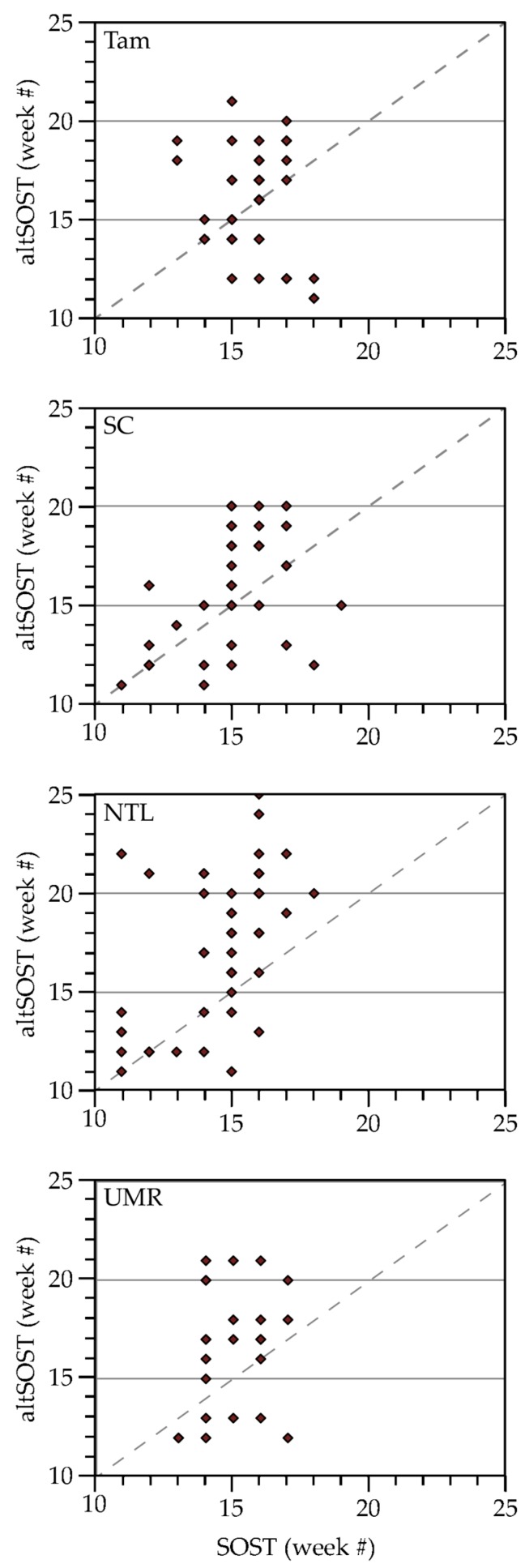
Comparison of the original metric for start-of-season timing (SOST) and our alternate metric (altSOST). The altSOST metric usually, though not always, delayed the perceived start of vegetation green-up. The diagonal dashed line references a 1:1 correlation. Tam = Tamarac National Wildlife Refuge. SC = St. Croix National Scenic Riverway. NTL = North Temperate Lakes Long-term Research Area. UMR = Upper Mississippi River floodplain.

SOST values indicated that new growth of vegetation occurred after the onset of GDU, but sometimes earlier than we would expect to observe leaf-unfolding, based on field visits and considering that soil warming (to enable plants to access water and nutrients) lags behind warming of air temperatures [68]. The altSOST metric typically (though not always) identified a later start to green-up than did the SOST metric (Figure 5) and was better correlated than the SOST metric with the onset of GDU (Table 2). The earliest SOST dates (week 11) for SC and NTL all occurred in 2010. The earliest dates identified for Tam and UMR (week 13) were equally split between 2010 and 2012. The altSOST metric identified the earliest dates for Tam as week 11 in 2012, week 11 in both 2010 and 2012 for SC and NTL, and week 7 in 2012 for UMR.

### 3.2. Seasonal Summaries

#### 3.2.1. Weather

The largest total GDU occurred in 2012 in all study areas (Figure 3). The ranking of total GDU, from high to low, for the other years we studied was 2010, 2011, 2008, and 2009, although the total GDU for Tam was similar in 2010 and 2011 (with the rate of increase somewhat faster in 2011). Notably, 2011 was a year of late snowmelt and a correspondingly late onset of GDU, but the cumulative GDU was still relatively high. 

The coolest growing-season temperatures occurred in 2009, which also was a year of low total precipitation from January through August (Figure 3). The relatively lower precipitation in 2009 might have been an extension of relatively lower precipitation after mid-July in 2008, which included a very light snowpack during the winter of 2008/2009. In contrast, 2012 was the warmest year, with relatively high rainfall totals in Tam, SC, and NTL. However, total rainfall in the UMR in 2012 was the lowest for the five years we studied. The highest total January–August precipitation occurred in 2010 in all study areas, which also was the second warmest year during our study.

#### 3.2.2. Seasonal ET Dynamics

**Figure 6:**
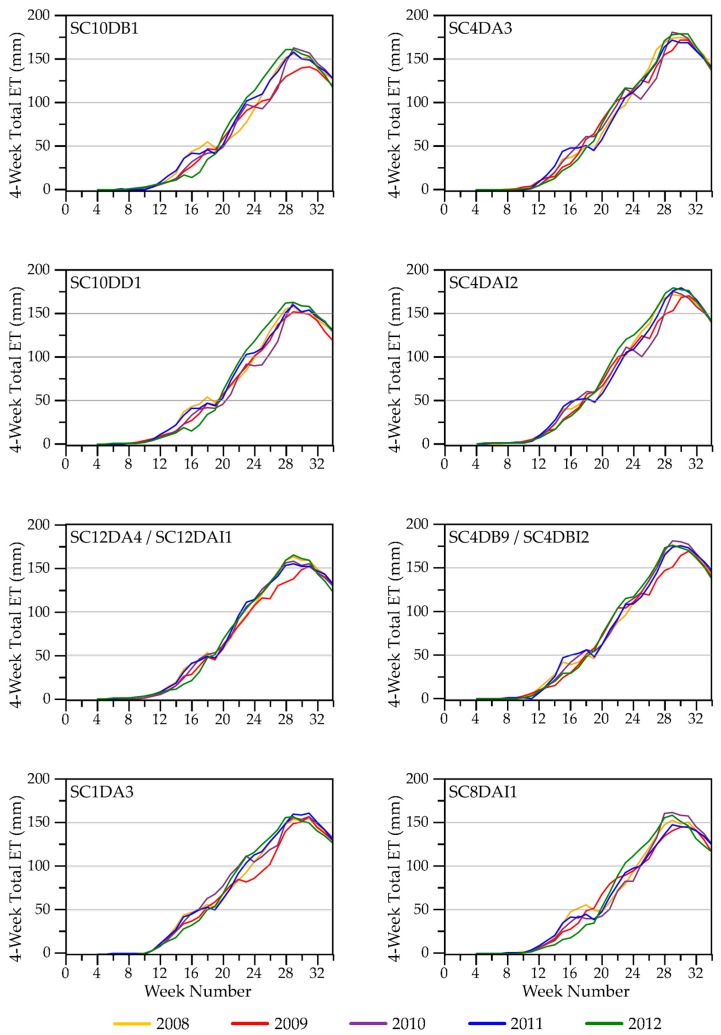
Four-week total evapotranspiration (ET), incremented weekly, for blocks in the St. Croix National Scenic Riverway study area. Sites SC12DA4 and SC12DAI1 occur within the same study block, as do sites SC4DB9 and SC4DBI2.

We compared seasonal ET dynamics with variation in weather variables across our study blocks. Four-week total ET, incremented weekly, for the SC blocks in Figure 6 is one example (see Figure S2a–c for seasonal ET trajectories for the remaining study areas). We used four-week totals to represent a standardized monthly time step similar to Walter’s construction of climatographs [69] and to recognize that plants (depending on species) can access water resources deeper in the soil profile [70], as well as at the surface, potentially representing precipitation inputs over several weeks or longer. Peak ET levels for 2009, the coolest, driest year (Figure 3), were lower across all study areas than in other years. We observed the highest peak ET values in 2010 and/or 2012, the warmest years. Temperatures were warm and rainfall occurred early in 2012, relative to the other years, but we did not detect an early season response in ET. Based upon weather-station data, we found that the previous year (2011) ended with drier than usual conditions, likely leaving low soil moisture at the beginning of 2012 (see Palmer Drought Severity Index [71,72] graphed in Figure S3). Spring rise in ET for most SC sites was earliest in 2008 and 2011 (Figure 6), the years with the latest snowmelt (Figure 2), and may have been due to ablation of the snowpack.

We compared block-level ET values with ET values for the individual pixel (native resolution = 1 km^2^) associated with each wetland site for 2008 to 2012 to evaluate whether results at the coarser scale altered the outcomes of our analyses. We observed a tendency for ET estimates at the block scale to be slightly higher than at the pixel scale, although Pearson correlation coefficients between blocks and pixels were very high for all study areas (Tam = 0.9950; SC = 0.9959; NTL = 0.9930; UMR = 0.9909). One of our motivations for using landscape blocks was to reduce the potential for persistent excessive water in pixels to inhibit the sensitivity of the SSEBop model to changes in landscape conditions. We used the National Land Cover Database (NLCD) thematic map for 2011 (pixel size = 30 m) to estimate the possible extent of surface water in blocks versus in pixels. We found that for 2011, the ET pixels at seven of 34 wetland sites had 50% or more area mapped as water or wetland. Similarly, seven of 33 landscape blocks contained at least 50% of their area mapped as water or wetland. Therefore, upscaling from pixels to blocks preserved the proportion of landscape area in water/wetlands while including some block pixels that represented sufficient upland area to exhibit variation in ET responses.

#### 3.2.3. Seasonal NDVI Dynamics

**Figure 7:**
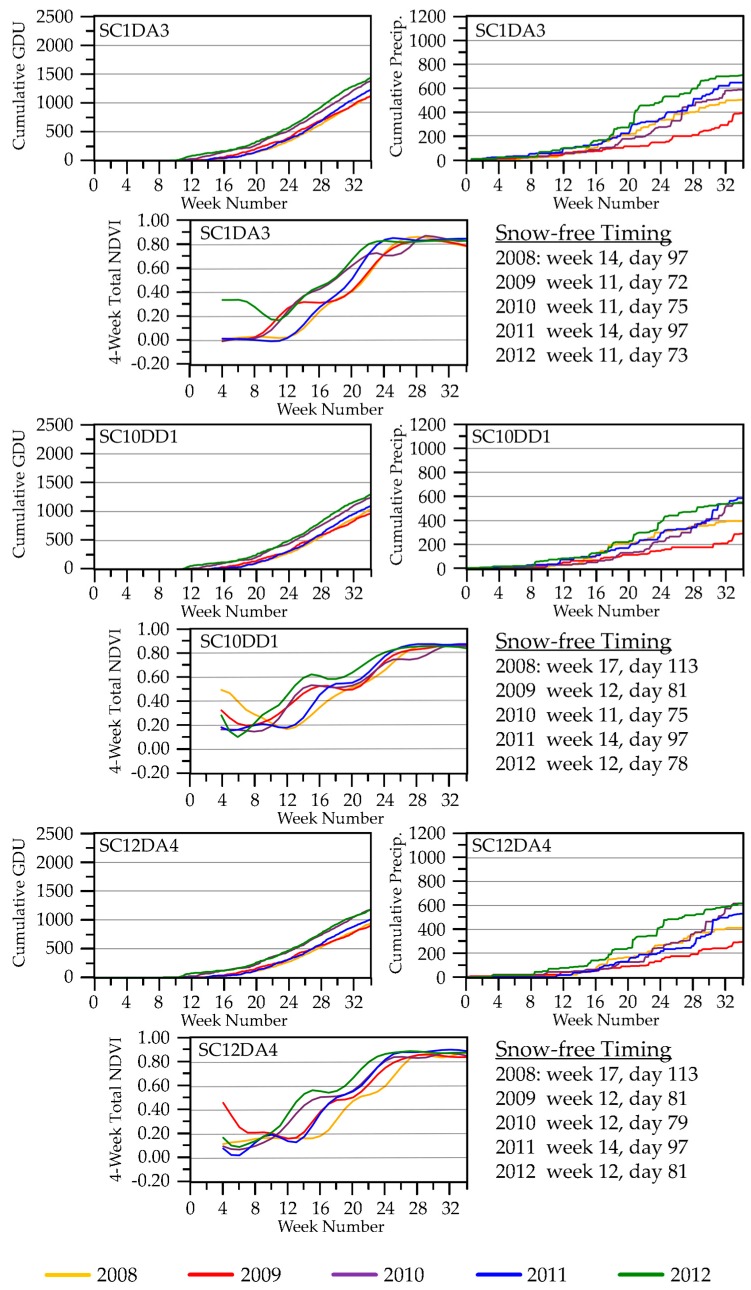
Cumulative precipitation and growing degree units (GDU) and four-week total NDVI from January through August for selected sites from the St. Croix National Scenic Riverway study area. NDVI was summed for each four-week interval, incremented weekly.

Within-season NDVI values aligned with coinciding cumulative precipitation and GDU (see Figure 7 for examples for some SC blocks; Figure S4a–d shows seasonal NDVI trajectories for all blocks by study area). We used four-week totals for the same reason described for ET. NDVI values indicated that green-up occurred earlier in 2012 than in other years, in association with warmer air temperatures. Late snowmelt delayed green-up in 2011 and, especially, 2008. Green-up progressed more slowly in 2009, a year with cooler air temperatures and drier conditions. We observed a very early increase in NDVI for a few SC sites (SC8DAI1, SC10DB1, SC10DD1) in 2008 as a result of snow melting then re-blanketing the landscape for another month. Such false green-up events also were apparent in other years when snowmelt briefly exposed dormant winter vegetation, including evergreens (for example, during 2012 at most SC sites and 2009 at SC10DD1 and SC12DA4).

NDVI values for blocks were highly correlated with NDVI values for pixels, but slightly less so than the correlations we observed between ET block and pixel values. However, ET pixels, sized at 1 km^2^, were closer in area to block size (4 km^2^) than were NDVI pixels (approximately 0.06 km^2^). Pearson correlation coefficients for NDVI block values versus pixel values were 0.9865 for Tam, 0.9829 for SC, 0.8981 for NTL, and 0.9512 for UMR. The proportion of times (from January through August) that NDVI values differed by more than 15% (a level we chose arbitrarily) between blocks and pixels was 1.0%, 0.9%, 13.0% and 8.8% for Tam, SC, NTL, and UMR, respectively. At UMR these differences always were due to NDVI values for blocks being lower than NDVI values for pixels, whereas we did not observe any directionality to such differences for the other study areas.

Seven of the 35 NDVI pixels associated with our field sites had more than 50% of their surface area mapped as water or wetland in the NLCD 2011 product (although, we note that wetlands may support dense stands of aquatic vegetation [73]). Several of those pixels were masked in the NDVI product because of perennial water cover. We used study blocks to help avoid data gaps from masked pixels, as blocks could provide NDVI information over a larger, but still local, area. As stated previously, seven of 33 landscape blocks also contained at least 50% of their area mapped as water or wetland; therefore, upscaling from NDVI pixels to study blocks preserved the proportion of potential wet areas in the landscape and enabled us to ameliorate the influence of data gaps from masked pixels.

#### 3.2.4. An Integrated Look at the Variables

**Figure 8:**
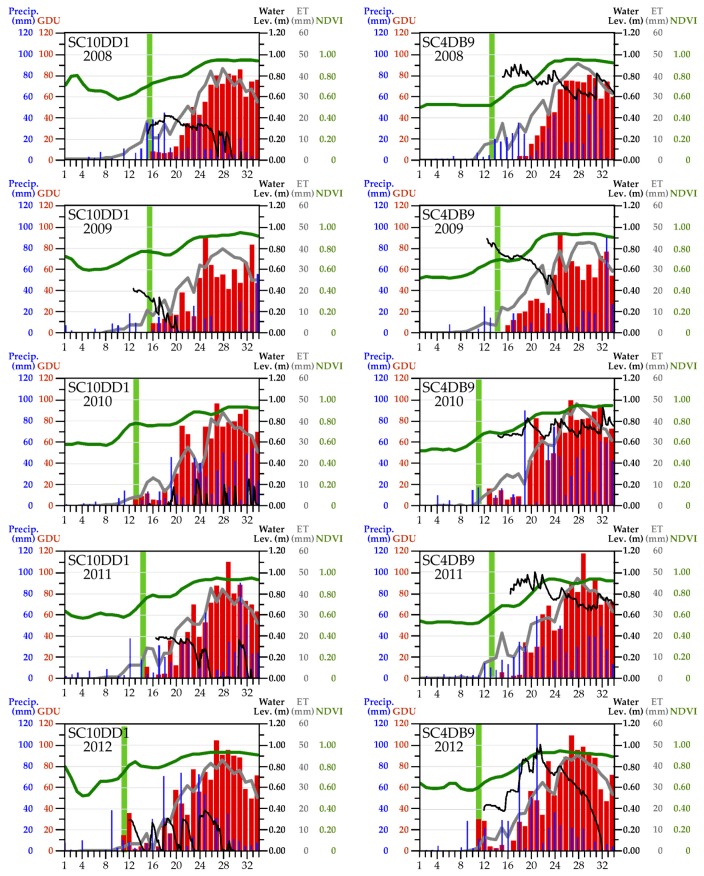
Overlay of seasonal measurements and events for sites SC10DD1 and SC4DB9 from the St. Croix study area. Red vertical bars depict weekly total growing degree units (GDU) and blue vertical bars depict weekly total precipitation (Precip) from weather station data. We show satellite-derived measurements with the green line for the weekly Normalized Difference Vegetation Index (NDVI) value and the grey line for weekly total evapotranspiration, the latter interpolated between data gaps for weeks 5, 13, 21, and 29 to render this graph (we did not use interpolated data in our analyses, though). The black line indicates the daily median water level measured by pressure sensors in the wetland at each site and the light green vertical bar is the interval in which we identified the first amphibian calls of the season from site acoustic recorders.

**Figure 9:**
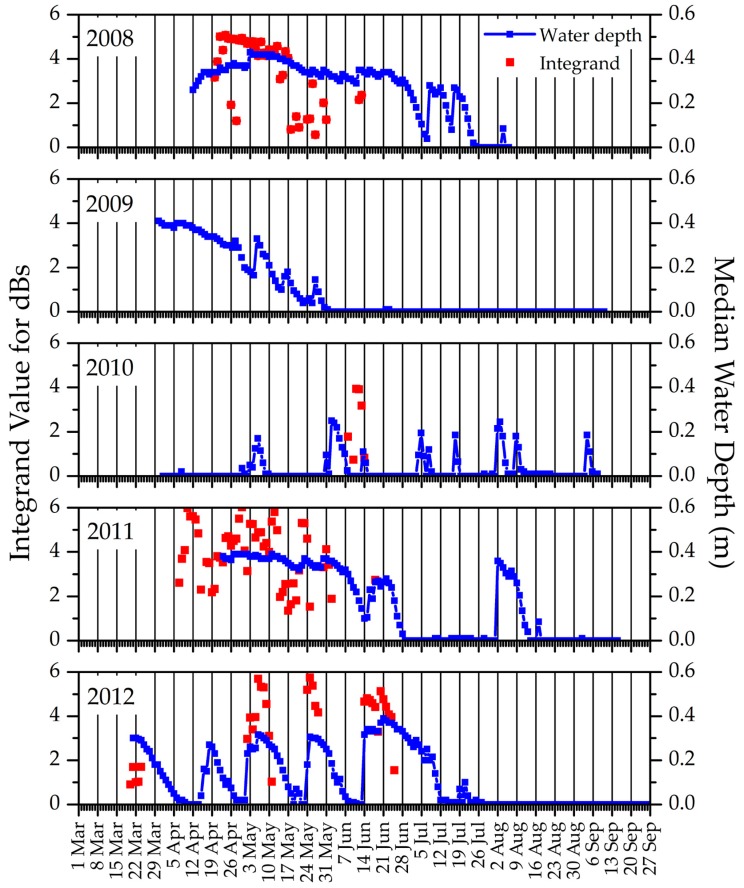
Amphibian calling activity during the reproductive season for 2008–2012 for *Pseudacris crucifer* (spring peeper), an early breeding frog species, compared with median daily water depths for site SC10DD1 in the St. Croix study area. Calling is represented as integrands (area under the curve) for daily sound intensity based on dB levels of *P. crucifer* calls recorded for five minutes every hour over the course of each season. *P. crucifer* did not call during dates lacking integrand values. Daily median water depths were calculated from hourly samples recorded on pressure loggers installed just above the sediments at the deepest location in the wetland.

We overlaid seasonal measurements and events identified from data we acquired in situ and by satellite to compare how they tracked with respect to precipitation and air temperatures. We show examples for two SC sites (SC10DD1 and SC4DB9) in Figure 8. ET activity (grey line) generally followed temporal patterns of GDU (red vertical bars), but additionally would have been affected by precipitation inputs (blue vertical bars) and, at the beginning of the growing season, timing and amount of snowmelt (not shown). These same factors would have influenced NVDI values (green line), as would the annual cumulative increases in leaf area from initial bud burst through peak season. Water levels (black line) behaved differently from one another at these two sites, with substantially more episodes of drying and rewetting over the study years in SC10DD1 in contrast with more consistency in SC4DB9. Amphibians typically starting calling (light green vertical bar) with the onset of warming temperatures; however, these temperatures did not necessarily need to be high enough to support plant growth (i.e., the minimum threshold required for GDU). Amphibian calling ceased when wetlands dried early, as shown in the calling record for *Pseudacris crucifer* (spring peeper) plotted against water levels recorded for SC10DD1 across the five years we studied (Figure 9).

## 4. Discussion

Few scientific data are available for understanding how changing climate actually is affecting key ecological conditions and processes on wetland-upland landscapes, such as those we studied in the U.S. Midwest. Yet, these data are necessary for resource managers and other stakeholders to understand the nature of real changes, not those simply predicted by risk models [21], and to manage resources effectively and adaptively in the face of climate change. This lack of critical information motivated us to design and implement a study integrated across scales, sensors, and disciplines to assess recent relations among temperature, precipitation, wetland and upland water availability, and the statuses of amphibian populations in our study areas [27]. Our analysis of the first five years of data collection provided an opportunity to further evaluate our approach for executing the remote sensing component of the assessment.

A limited number of previous reports have described long-term studies with integrated remotely sensed and ground measurements collected over a variety of sensors and scales [74,75,76,77,78]. These efforts illustrate various considerations in selecting which variables to measure and in determining approaches to summarize the variables in meaningful and complementary ways. Clearly, the scientific questions being addressed largely dictate the types of measurements that are necessary to ultimately conduct effective analyses, but selecting the best metrics can be complex and nuanced. For example, measurements of air temperature are key to climate-related research, but could be analyzed as individual observations at some periodicity, averages per unit time, high or low readings per unit time, or cumulative observations or derivatives (such as GDU). As White et al. [62] demonstrated for start-of-spring metrics calculated with NDVI, no single metric necessarily works best under all circumstances or in all geographic settings, as illustrated by the range of metrics chosen by researchers studying effects of climate change in different geographic areas or on different taxa or species (e.g., [79,80,81,82]). 

Relations of precipitation and water availability are crucially important to measure for assessing climate’s effects on biota. However, studying wetland-rich landscapes via satellite sensors is challenging because water presence can be highly dynamic and also amplify ambiguity in spectral signatures of land cover measured by such sensors. For example, drought typically will depress NDVI values, but so will too much water on the landscape, even if plant growth is vigorous, because water absorbs energy in the portions of the spectrum used to derive NDVI [83]. The presence of water can especially complicate the optical signal in sensors like MODIS with wide optical bandwidths. Using remote sensing to determine water availability in wetlands in wooded settings is particularly difficult because most wetlands are small, relative to sensor resolutions, and are obscured by overstory vegetation [84]. Thus, wetlands are notoriously difficult to map with consistent and high accuracy [84] (e.g., see results in [85,86,87,88,89,90]) and their high spectral, spatial, and temporal variability present special challenges for phenological studies [91]. Recent mapping products and/or algorithms provide new opportunities to characterize the presence and dynamics of water in the landscape [17,18,92,93,94] by capitalizing on water’s distinctive signature across parts of the energy spectrum used by remote-sensing scientists to monitor changes on the Earth’s surface [83,95]. Yet, these new advances are best suited for moderate-sized or larger wetlands or bodies of open water, and mapping small to midsized waterbodies and wetlands accurately at ecologically meaningful time scales remains difficult [84]. The release of the Global Surface Water Explorer [18,96] is advancing our understanding of water dynamics globally over the past three decades, but the time steps of the underlying Landsat data are irregular, in contrast to the consistent seven- and eight-day products we used, and results presented in the Global Surface Water Explorer typically pertain to waterbodies that are larger than the wetlands we studied and that are not obscured by the overstory and/or emergent vegetation that shrouded many of our study wetlands. For all these reasons we used variables that did not rely directly on remotely observing the dynamics of surface water, but rather on vegetation responses and ET activity as proxies for water availability over time.

### 4.1. Onset of Growing-Season Conditions

The timing and duration of the transitional period from winter to spring weather is important because they drive the timing of events for individuals of a species, as well as for the food sources and biotic and abiotic conditions that ensure the availability of suitable habitat required by those individuals. Early breeding amphibians in the U.S. Midwest, for example, require breeding wetlands to remain sufficiently warm once thawed, with ample water availability and suitable food sources to allow successful breeding, hatching, larval development, and metamorphosis to occur sequentially over a season. Unusually early winter thaws, followed by freezing temperatures or insufficient precipitation to sustain wetland water levels, can reduce the fitness of breeding amphibians that entered wetlands early in response to warm temperatures and snowmelt [43]. We used satellite-derived variables to try to detect the onset of spring conditions within landscape blocks as an indicator of when warm temperatures and snowmelt triggered changes in conditions across these larger areas, all in relation to what we observed at our individual study wetlands. 

Given plants require suitable combinations of temperature and water availability, along with sunlight, to photosynthesize and grow, we considered the ET variable we used as a functional indicator of such conditions across landscape blocks, especially water availability when temperatures and insolation were above physiological thresholds [97]. We observed interannual differences in total monthly ET during spring months, but the timing of initial ET activity was not sensitive to the timing of the onset of GDU. The onset of ET activity, as we defined it, frequently began during the same week from year to year at a given site, regardless of differences in air temperatures or snow presence. This may have been because ET estimates likely included noise from random errors caused by parameterization of the SSEBop model, as well as poor data quality from cloud contamination in the land surface temperature data. The relative impact of such errors tends to be higher at small ET estimates, such as during the early part of the growing season. Therefore, it may be appropriate for us to alter the way in which we define the onset of ET activity.

We used NDVI as an indicator of vegetation responses to seasonal temperature and precipitation dynamics within landscape blocks, particularly for the timing of spring green-up. Prior studies have highlighted the strengths and weaknesses of NDVI for estimating the onset of the growing season and have shown variability in accuracy of estimates depending on plant functional types [98]. For example, the extraction method used to generate the SOST metric compared favoriably with others tested for estimating the start-of-season over temperate deciduous forests and grasslands [62], but as Hmimina et al. [99] pointed out, using MODIS-derived NDVI in evergreen-dominated landscapes may not provide consistent nor detectable phenological patterns. We found that the onset of greenness indicated by the SOST metric appeared at times to be influenced by false local minima and maxima in NDVI response curves that probably were related to pre-season snow-on/off conditions, especially in settings with evergreens [100]. This resulted in some SOST dates that likely indicated pre-season exposure of dormant vegetation, rather than onset of green-up. For example, the SOST metric for Tam site TA1DB1 in 2009 estimated the onset of green-up in Week 16, around mid-April, whereas the altSOST metric indicated onset in Week 18, the beginning of May. We visited that site in mid-May of 2009 and observed the early stages of leaf-out, which could have been underway for two weeks (i.e., Week 18), but likely were not underway during the middle of April (i.e., Week 16; see photos in Figure S5).

We selected NDVI for studying phenology because it is straightforward to calculate and has been, by far, the vegetation index most frequently applied for phenological studies [25,39,62,101,102,103]. NDVI has been shown to agree well with the start of carbon uptake by plants [104]. However, the Enhanced Vegetation Index (EVI) has been gaining popularity since MODIS data became available in 2000. EVI remains sensitive to canopy variation even at high levels of leaf area where NDVI has been to shown to saturate [105,106,107,108]. EVI-derived start-of-season estimates have been better correlated with ground-based start-of-season in deciduous forests, especially in the eastern and northern United States; however, in our study region, green-up dates estimated from NDVI were significantly correlated with those estimated from EVI [107]. Thus, we believe only modest improvements in identifying SOST for our study areas might be gained with EVI.

As others have stated, accounting for winter background levels of NDVI is important for avoiding false green-up signals [100]. To address this we estimated altSOST for each landscape block based on a threshold representing the study area winter background level of NDVI averaged across each area’s individual blocks. In retrospect, we might have observed greater concordance between the start of green-up and GDU if we had used background NDVI levels specific to each landscape block, as the averages we used could introduce noise based upon study-area-wide land-cover heterogeneity. Winter NDVI values were quite high at some of our sites (such as those shown in Figure 8), but not at others within the same study area. Using altSOST helped us reduce the influence of false green-up signals when we were trying to identify the start of growing conditions, but a more refined SOST algorithm that could directly, or by some proxy, locally self-calibrate for winter background conditions potentially would be more effective. 

Researchers have various perspectives on relations between snow cover/snowmelt and land-surface phenology [109]. Snow is considered by some as a source of noise that introduces error in phenology estimates [110,111]. Others have recognized that the presence of snow (e.g., snow moisture equivalent) can be strongly related to the timing of the start of the growing season [112]. For example, NDVI values generally will rise during snowmelt, and this rise may or may not coincide with a similar increase in actual vegetation activity [113,114]. Bottcher et al. [114] found close correspondence between the beginning of photosynthetic activity (from in-situ CO_2_ fluxes) and the timing of snowmelt as the ground in coniferous forest began to be exposed. Results by Sadinski et al. [27] suggest it is difficult to ignore snow/snowmelt in studying land-surface phenology, as they found green-up to be closely aligned with the timing of snowmelt in years when snow melted later, but not in years when snow melted earlier. Ultimately, we need an improved method for addressing the influence of snow on our ability to detect the onset of green-up, as a good estimate for SOST is critical not only for studying changes in weather/climate conditions in relation to biological responses at the start of the growing season, but also because SOST is used in calculating additional phenological metrics, such as time-integrated NDVI and the length of the season [25]. 

The snow product we used was composited at eight-day intervals, a coarser time step than we preferred for identifying the start of growing conditions. The snow dataset was accompanied by binary information about daily snow presence during each of the eight days in a composite interval. However, a “1” indicated that snow was detected on a given day, but “0” did not distinguish between snow being absent and clouds or other interference obscuring the Earth’s surface from the sensor. NASA did offer a daily snow product [63], which conceivably would have allowed us to describe snow presence more accurately, but the volume of daily data was beyond our capacity to process them. The resolution of the snow product we used also was spatially coarse relative to the ability of amphibians to move across the ground to access breeding wetlands when snow cover was spatially discontinuous [27] within a “snow pixel.” It therefore was reasonable to have detections of snow on the ground during an eight-day period in which amphibians also called for the first time that season.

### 4.2. Tracking Growing-Season Conditions

#### 4.2.1. Seasonal ET Dynamics

At the block level we analyzed dynamics of water availability within and across growing seasons. ET exhibited interannual differences (e.g., Figure 6 and Figure S2a–c) that we could relate generally to seasonal differences in precipitation and air temperatures among years. For example, ET for SC was lower in years that were drier and/or cooler and higher in years that were wetter and warmer (Figure 6, with reference to SC in Figure 3). ET values were relatively more similar among years for the other three study areas, but for differing reasons. Tam sites (Figure S2a) likely had more consistent supplies of shallow groundwater [115,116], which, other than in 2012, may have helped maintain water in wetlands and moisture in the rooting zones of upland plants. NTL (Figure S2b) had more evergreen needleleaf vegetation than occurred in the other study areas, which provided more consistent vegetation cover than afforded by deciduous vegetation or grasses. UMR blocks (Figure S2c) included high proportions of standing water, which could moderate the detection of changing conditions in the upland areas of the study blocks. 

Interpreting the status of moisture in our study landscapes via ET activity is less straightforward than interpreting the direct presence of standing water. For example, the reduced presence of standing water typically means reduced moisture in the landscape, which could result in lower ET; but ET activity also could lessen because of cooler air temperatures or reduced insolation. Thus, using ET to assess water conditions requires that we consider the interplay among current and recent surface temperatures, moisture inputs, and insolation, as well as local land cover. The strength of a variable like ET, however, is that it does integrate multiple facets of the environment (ambient environmental conditions, land cover, and vegetation condition, life history, and phenological stage) and can provide a relatively more holistic perspective than any of its individual component variables.

The SSEBop model that produced the ET data was developed mainly to quantify irrigation water use, where there is a clear contrast in surface temperature between irrigated and non-irrigated lands. SSEBop’s application in more water-rich areas can challenge model parameterization and interpretation. SSEBop is a two-parameter model that creates an ET fraction from land surface temperature by expressing the relative magnitude of land surface temperature between a lower and upper limit. The lower limit (cold/wet) is determined using a vegetation index (NDVI) to identify well-watered vegetation that transpires at the maximum rate. Finding such pure pixels for calibrating SSEBop in water-rich areas is challenging because the water signal reduces the NDVI. This is particularly true early in the growing season, when it is hard to find pixels with high NDVI (>0.7). In such cases, SSEBop will be calibrated using NDVI from locations that are farther from the study area, which can introduce uncertainty in the model calibration. Cloud contamination and haze also can interfere with accuracy of SSEBop’s performance. Although the model is capable of detecting clouds because of their extreme cold temperatures, haze and partial cloud contamination will introduce errors in the estimation of land surface temperature and, thus, ET. The end result is an exaggeration of ET from cooler land surface temperatures as a result of clouds or from limited emitted radiation reaching satellite sensors because of haze [117]. Issues with geometric registration of the MODIS thermal data (spatial resolution = 1 km^2^) can further influence the quality of the ET data. Haynes and Senay [118] identified a spatial shift and mismatch between NDVI and land surface temperature from MODIS by as much as 2 km. Additionally, the coarse size of the thermal pixels mean that SSEBop is responding to a mix of signals coming from the landscape. Such mixed pixels, along with source-data misregistration, make it more difficult to parameterize the wet/cold lower boundary of SSEBop well and further complicate interpreting ET results with respect to landscape properties and responses. Challenges like these can be addressed using datasets with higher spatial resolution, such as from Landsat, which offers a thermal signal approximately 100 times finer than that of MODIS, but the tradeoff is a lower repeat interval for observations. 

#### 4.2.2. Seasonal NDVI Dynamics

NDVI exhibited a wider range of annual responses than did ET to changes in temperature and moisture conditions. This could have been because NDVI was computed directly from a relation between two bands of the energy spectrum measured by a single sensor, whereas ET was estimated from several sources of remote measurements and an energy-balance equation and, thus, included more inherent error. We found that the NDVI phenological curves were relatively easy to interpret (Figure S4a–d), given the temporal characteristics of snow-on/off (Figure 2) and dynamics of precipitation and growing degree temperatures (Figure S6a–d). The NDVI seasonal trajectories in Tam, for example, began to rise once snow was gone and air temperatures were sufficient for plant growth. Late snowmelt delayed green-up in 2008 and 2011, whereas cool temperatures likely delayed green-up in 2009 (a year with early snowmelt). These differences in environmental conditions corresponded with phase offsets we observed in growth curves across the five years (Figure S4a). The Tam blocks also revealed interesting within-season characteristics among the NDVI trajectories. The years 2012 and 2010 were the first and second warmest growing seasons, respectively, in the historical climate record dating back to 1895 [72]. NDVI response curves ascended earliest in those two years. Interestingly, the 2010 curve started to descend around week 22 (beginning of June), during a part of the season when NDVI responses in other years were hitting peak greenness. This could have been due to Tam receiving only average precipitation in 2010 while cumulative evapotranspirative demand was high. A boost in precipitation starting around week 26 (beginning of July) that year was associated with an uptick in the NDVI curve towards peak greenness and ultimately contributed to 2010 being the wettest growing season of the five we studied. In contrast, wetlands we sampled in situ in Tam did not dry (with one exception) prematurely in 2010. Their water levels presumably were maintained by underlying shallow groundwater [115,116], but water levels did increase from the boost in precipitation that began in July [27]. Wetland hydroperiods were relatively short in Tam during 2012, the warmest year on record, when precipitation after week 32 (mid-August) was limited. The Tam sites already had reached peak greenness by that time in 2012, so the influence of drying conditions on NDVI was more subtle than in 2010.

### 4.3. Other Considerations

We knew that the native pixel resolutions of the satellite-derived products available to us for this study would preclude us from observing detailed spatial and temporal dynamics of ET and NDVI within our landscape study blocks. Thus, we had to consider important nuances inherent in analyzing remotely sensed data, such as how best to analyze data associated with different pixel sizes across a given space, to try to maximize the ecological relevance of the remote-sensing outcomes to the finely detailed biotic and abiotic data we were collecting on the ground at individual study wetlands. Though it might appear paradoxical that we chose a block size that was four times larger than our coarsest satellite-derived product, we did so primarily because a spatial expanse of 4 km^2^ was relevant ecologically for the amphibians that reproduced in our individual study wetlands [43], as well as for many other species that inhabited our study areas. Also, surface-water availability, a key variable we measured in our individual study wetlands located within the blocks, is linked inextricably to physical, vegetative, and edaphic factors on the surrounding landscape [119]. We hypothesized that measures of ET and NDVI across a surrounding, 4 km^2^ area would reflect changes relevant for surface-water availability in our individual wetlands, and vice versa. Given resources did not allow us to sample more wetlands intensively within blocks, the remotely sensed data for each block provided us complementary and spatially continuous observations across seasons, which enabled us to describe a broader context for interpreting how weather and climate conditions were affecting water availability and the statuses of amphibian populations across landscape matrices of wetlands and uplands within our study areas [27].

The different data we used were produced at different time steps. Weather-station data were daily summaries; snow and ET data were composited at eight-day intervals; and NDVI data were composited over seven-day rolling intervals. We measured surface-water levels and amphibian calling activity hourly at individual study wetlands, which we typically summarized at a daily scale [27]. After considering these various time frames, we decided to standardize all the remotely sensed variables and associated summaries of weather-station data to seven-day intervals, beginning with the first day of January each year. We accepted that we would have certain weeks with no assigned eight-day products (see Table A4), but chose that option over expunging some of the NDVI data to accommodate the coarser, eight-day time step of the ET and snow data. The source data for the remote products originated with daily acquisitions by MODIS sensors, but it was beyond the scope of our resources to investigate to what extent daily data could have improved the temporal sensitivities of our analyses. We might have realized improved precision on pinpointing the timing of weather-related events, but we also would have inherited the cloud contamination that the composited products overcame. We hypothesize that the overall shapes of the seasonal trajectories for ET and NDVI generated from daily data would have been comparable with those we generated from the composited products. 

Using remotely sensed data for long-term studies implies working across satellite sensors and/or data collections. In our case, we used remotely sensed products generated with Collection 5 data from MODIS. The Collection 5 data are known to have radiometric instability and sensor degradation affecting the red and NIR channels, and thereby time-series NDVI [120,121,122]. However, Collection 5 data were the best available observations at the time of our study and, although our analyses may have been impacted by MODIS sensor degradation apparent after 2007, we were able to identify logical relations between the Collection 5-derived variables and those measured at weather stations. Global historical Collection 6 MODIS data became available shortly after we completed our analyses and will provide an improved source of observations and products for our future analyses.

The satellite-based data gave us spatially continuous information across our study areas, making it easy to co-locate remotely sensed data with field sites. We did not have such flexibility with the locations of long-term weather stations, which were not positioned within close proximity to some study blocks. Although our comparisons of temperature and precipitation data that we measured at some individual wetlands showed they were similar to data we obtained from the nearest weather station, data from the more distant stations might have introduced noise into some of our results, particularly for precipitation totals. This potentially was most problematic for Tam, where the closest continuous long-term weather record for any of the study blocks was from a single station 20–35 km away. Assuming a network of several weather stations for each study area is not realistic, improvements to future analyses might result from using spatially continuous surfaces generated from weather-station data for climate variables, such as those produced by the Daymet model [51,52]. Our analyses were limited to data from stations that provided continuous observations over the period of our study, but Daymet, which builds surfaces of 1 km^2^ spatial resolution using data from a network of stations, can incorporate information even from stations with shorter periods of observation. So the resultant products from Daymet are based upon a richer data record than we could accommodate with our approach. Our cursory assessment indicated Daymet precipitation and temperature surfaces looked reasonable in comparison with data from surrounding weather stations.

Climate-related remote-sensing or modeling studies commonly use total or cumulative precipitation, but researchers have used a wider variety of ways to summarize air temperature (e.g., [79,123,124,125,126,127,128,129,130,131]). We used GDU calculated with average daily air temperatures, which we then cumulated over the growing season or used as interval-based events that potentially were relevant as a trigger for the onset of plant growth. Alternatively, Piao et al. [132] suggested that leaf unfolding dates correlated well with maximum daily temperatures, a metric also incorporated by others [133].

We assessed ET and NDVI for all landscape blocks within a study area without regard for differences in cover types. Within blocks, mixtures of cover types can challenge successful pinpointing of phenological events [134]. Even differences in microclimate can cause substantial spatial variability in the timing of leaf onset within distances less than 100 m [135]. Results from past studies have provided ample evidence that different cover types, indeed species, respond differently to changes in water availability in the landscape (e.g., [136,137,138,139,140,141]), and heterogeneity in vegetation may retard detection of drying conditions, as different species can access soil moisture from different depths in the rooting zone after surface water has dried in wetlands [142,143,144]. We could expand our future analyses to investigate the degree to which ET, NDVI, and the duration of snow cover are associated with land-cover types in our study areas.

Studying relations between land-cover types and Earth-surface phenology could be more easily accommodated by employing time-series data from satellite sensors that provide finer spatial resolution than data from MODIS. Coarse-scaled spatial data from daily satellites, such as MODIS, typically have been the foundation for time-series analyses of changes in land-cover conditions associated with climate/weather [145,146]. Sensors having relatively finer resolution, such as Landsat’s 30-m pixels, have not provided adequate temporal frequency for these analyses. However, recent technological advances make it feasible to compile dense time series of clear observations with Landsat data at the pixel level [147]. Such data, although not providing as consistently frequent temporal observations as daily sensors like MODIS, can be used to assess changes in vegetation conditions that relate to climate/weather [145]. Investigators also are testing whether enriching Landsat time-series data with harmonized Sentinel-2 data can further extend this capacity [148]. Calculating annual phenological transitions operationally with data from Landsat or other moderate-resolution sensors requires overcoming computational and data-frequency hurdles, but progress in this area would go far towards addressing issues related to land-cover heterogeneity for both NDVI and ET.

We evaluated ET activity, vegetation response, and snow presence as proxies for landscape moisture availability and other growing conditions and analyzed these derived variables with respect to precipitation and air temperatures within replicate 4 km^2^ blocks in four study areas. These efforts were part of an integrated study that also included analyses of relations of precipitation and air temperature to wetland surface-water availability and amphibian calling activity that we measured via ground-based sensors at individual wetland study sites [27]. The ET and NDVI data we analyzed, with the mix of land-cover features they represented, gave us perspective on the conditions of the landscape at large, which showed a modest range of differences in responses from year to year over the period of our study. Responses to changing conditions were more dynamic at the scale of the individual wetlands we studied from the ground (e.g., Figure 8). At that local scale, critical changes in conditions had potentially dramatic consequences for amphibians when our study wetlands dried (Figure 9). The concurrent cessation of amphibian calling (as a proxy for breeding activity) could indicate the likelihood of not having embryos and/or not having sufficient time for embryos to develop fully and metamorphose in a given year, impacting population recruitment and persistence. Changes in water availability required more time and had to be more pronounced before they were apparent in the mix of wetland and vegetation types aggregated at the scale of landscape blocks. This is important because, although satellite remote sensing can provide global information over a wide range of scales in a consistent and repeatable manner [22], our findings underscore the importance of integrating remotely sensed data with data collected on the ground [149] to reduce the risk of missing events or changes in conditions that could threaten biodiversity.

## 5. Summary and Conclusions

We retrospectively asked whether decisions and assumptions we made in applying remotely sensed data for a large, multiscale, integrated study on changing environmental conditions in wetland-rich landscapes were appropriate for maintaining the strength of relations among variables and for judging their suitability to track and understand change. Mapping wetlands and water with remotely sensed data has historically produced inconsistent and unreliable results, especially in wooded landscapes. Therefore, we evaluated if ET and NDVI were suitable proxies for monitoring landscape-level moisture. We also tested whether these variables were good indicators for the onset of growing-season conditions, as indicated by GDU and snow-off, and whether the definitions we imposed for snow-off and start-of-season were appropriate.

We found that the ET variable exhibited a small range of interannual differences that we could relate to seasonal patterns of precipitation and air temperatures. However, the ET responses likely were variously tempered by available shallow groundwater supplies, presence of evergreen vegetation, and/or high proportion of standing water in the landscape. ET activity clearly increased from early through late spring, but the ET variable was insensitive to the timing of spring onset of GDU that would drive evaporation and foliar growth (and respiration). This may suggest that we should adjust the way in which we define the onset of ET activity. ET, as a variable that integrates multiple environmental factors, is not straightforward to interpret for an application like ours.

NDVI exhibited a greater range in interannual responses to precipitation and air temperatures, was fairly simple to interpret, and was sensitive to the timing of the onset of GDU. Yet, we observed the SOST algorithm that identified the start of vegetation green-up was not robust to false green-up events caused by winter snowmelt, particularly in settings with evergreen vegetation. We therefore defined an alternate start-of-season variable (altSOST), calibrated by winter background levels of NDVI, which resulted in a stronger correlation with the GDU record. From this we concluded that the original SOST algorithm needs further refinement to better handle winter maxima/minima cycles in the NDVI response curves. 

We used satellite-derived products that imposed constraints on the temporal domain of our analysis. These products compiled information over a standard eight-day time step (ET and snow), a rolling seven-day time step (NDVI), and a daily time step (SOST). The data we acquired from weather stations were daily records. Selecting a common unit of analysis to overcome these mismatched temporal resolutions required opting for data reduction to accommodate the coarser time units (e.g., via summarizing across observations or removing records) or data gaps to accommodate the finer time units (e.g., Table A4 shows there were no eight-day intervals matched to weeks 5, 13, 21, and 29). We chose the latter option to preserve the full complement of data for the NDVI response curves.

The satellite-derived products presented a range of native spatial resolutions, including 250 m (NDVI, SOST, altSOST), 500 m (snow), and 1000 m (ET) raster cells. In considering a common spatial unit of analysis, we were cognizant that pixels over areas with lots of standing water (e.g., in wetland-rich landscapes) can reduce the information value of ET and NDVI. We chose a spatial unit of analysis (2 km × 2 km) that was informed by reported home ranges and movement distances of amphibian species known to inhabit the areas we studied. This provided an ecologically meaningful basis for selecting the size of the study blocks, with each block encompassing a combination of wetlands and uplands that individual amphibians might encounter during a year. ET, NDVI, and snow-off dates calculated at the block scale were highly correlated with values from the specific, within-block pixels associated with locations of the wetlands we monitored on the ground. From this we concluded that landscape blocks preserved the information value of the native raster cells while ensuring that a mix of wetland and surrounding upland responses was represented and that data gaps were avoided from perennially inundated pixels that were masked in the original NDVI product. This outcome suggests we may feasibly reduce data storage and processing demands while providing ecologically meaningful units that retain the information value of the variously scaled source data.

We developed and tested a definition for seasonal snow-off based on criteria that made environmental sense for the north-central part of the United States that encompassed our study areas. We concluded that our definition was appropriate, but it likely has limited portability to other regions with different climate regimes. Ultimately, we need a more locally or regionally calibrated way to define snow-off that can be applied across the continent.

Overall, we found that landscape responses determined with data from satellite sensors generally resembled conditions measured on the ground at wetland sites and nearby weather stations. However, environmental changes were much more dynamic and dramatic on the ground than what we detected at the landscape scale. This means that conditions that put animal populations at risk on the ground may not be detected by satellite sensors or may be detected too late to alert resource managers and other stakeholders. This is a caution that global remote monitoring of the landscape is not sufficient by itself for identifying changes in conditions that put ecosystems at risk and underscores the importance of continued integrated, multiscale, and multiplatform studies. 

## Figures and Tables

**Table 1:** Timing of snow-off. (**a**) Frequency that snow recurred after the first single (post-February) snow-free interval from 2008–2012; (**b**) Frequency that snow recurred after the first two-week (post-February) snow-free interval from 2008–2012; (**c**) Comparison (Pearson correlation coefficient) of snow-free timing at the scale of cells versus blocks.

Study Area	(a) Proportion (and Percentage) of Sites Where Snow Recurred after One Snow-Free Interval	(b) Proportion (and Percentage) of Sites Where Snow Recurred after Two Snow-Free Intervals	(c) Correlation between Timing of Snow-Off at the Cell vs. the Block Scale
Tam ^1^	18/50 (36%)	2/50 (4%)	0.98
SC ^2^	10/50 (20%)	0/50 (0%)	0.99
NTL ^3^	9/50 (18%)	2/50 (4%)	0.97
UMR ^4^	7/25 (28%)	0/25 (0%)	0.93

^1^ Tam = Tamarac National Wildlife Refuge. ^2^ SC = St. Croix National Scenic Riverway. ^3^ NTL = North Temperate Lakes Long-term Research Area. ^4^ UMR = Upper Mississippi River floodplain.

**Table 2:** Comparison (Spearman rank correlation) of original start-of-season timing (SOST) and alternate start-of-season timing (altSOST) with the first week that median daily growing degree units were ≥1.

Study Area	SOST	altSOST
all areas	0.2076	0.4271
Tam ^1^	−0.0011	0.6456
SC ^2^	0.1701	0.3053
NTL ^3^	0.3399	0.5007
UMR ^4^	0.1571	0.3722

^1^ Tam = Tamarac National Wildlife Refuge. ^2^ SC = St. Croix National Scenic Riverway. ^3^ NTL = North Temperate Lakes Long-term Research Area. ^4^ UMR = Upper Mississippi River floodplain.

## Supplementary Materials

The following are available online at http://www.mdpi.com/1424-8220/18/3/880/s1, Figure S1: Interannual differences in early season monthly evapotranspiration (ET), Figure S2: (a) Four-week total evapotranspiration for blocks in the Tamarac National Wildlife Refuge study area, (b) Four-week total evapotranspiration for blocks in the North Temperate Lakes Long-term Research Area study area, (c) Four-week total evapotranspiration for blocks in the Upper Mississippi River floodplain study area, Figure S3: Palmer Drought Severity Index (PDSI) for the NOAA climate division (WICDO1) representing the St. Croix National Scenic Riverway study area, Figure S4: (a) Four-week total Normalized Difference Vegetation Index for blocks in the Tamarac National Wildlife Refuge study area, (b) Four-week total Normalized Difference Vegetation Index for blocks in the St. Croix National Scenic Riverway study area, (c) Four-week total Normalized Difference Vegetation Index for blocks in the North Temperate Lakes Long-term Research Area study area, (d) Four-week total Normalized Difference Vegetation Index for blocks in the Upper Mississippi River floodplain study area, Figure S5: Location near field site TA1DB1 in the Tamarac National Wildlife Refuge study area showing early and full stages of leaf-out, Figure S6: (a) Cumulative precipitation and growing degree units from weather stations we used to represent the Tamarac National Wildlife Refuge study, (b) Cumulative precipitation and growing degree units from weather stations we used to represent the St. Croix National Scenic Riverway study area, (c) Cumulative precipitation and growing degree units from weather stations we used to represent the North Temperate Lakes Long-term Research Area, (d) Cumulative precipitation and growing degree units from weather stations we used to represent for the Upper Mississippi River floodplain study area.

## Appendix A

Weather stations we used to determine growing degree units and precipitation input for landscape blocks in the four study areas.

**Table A1:** Weather stations we used to represent temperature and precipitation conditions for landscape blocks in the four study areas. In the first column, “primary” refers to a weather station from which we obtained the majority of weather data for a given study block and “mitigation” refers to weather stations we used to replace missing or questionable data from primary station. We did not use a secondary station when data sets from primary stations were sufficient. Tam = Tamarac National Wildlife Refuge. NWS = National Weather Service. ID = Identifier. RAWS = Remote automated weather station. MN = Minnesota. Tmin = Minimum daily temperature. Tmax = Maximum daily temperature P = Precipitation. KDTL = Detroit Lakes Airport-Wething Field. WS =Weather Underground station. AWOS = Automated Weather Observing System. SC = St. Croix National Scenic Riverway. NOAA = National Oceanic and Atmospheric Administration. GHCND = Global Historical Climatology Network Daily. WI = Wisconsin. KRZN = Burnett County Airport. KHYR = Sawyer County Airport. ASOS = Automated Surface Observing System. NTL = North Temperate Lakes Long-term Ecological Research site. KARV = Lakeland Airport/Noble F. Lee Memorial Field. UMR = Upper Mississippi River.

Study Area	Study Blocks	Weather Station; Type; Location	Latitude Longitude ^1^	Records with Missing/Questionable Data and the Mitigation Steps Taken
Tam (primary)	All Tam sites	NWS ^2^ ID 212201; RAWS; Detroit Lakes, MN	46.84889 −95.84639	Tmin, Tmax, and P missing for 8–16 April 2009. We substituted with data from Detroit Lakes, MN (station KDTL).
Tam (mitigation)	All Tam sites	WS ^3^ ID KDTL; AWOS; Detroit Lakes, MN	46.83 −95.89	
SC (primary)	SC1DA3	NOAA ^4^ ID USC00212881; GHCND; Forest Lake, MN	45.3397 −92.9125	None.
SC (primary)	SC4DA3 SC4DAI2 SC4DB9 SC4DBI2	NWS ID 470602; RAWS; Lind, WI	45.73972 −92.79556	Tmin and Tmax missing for 9 April–3 May 2008. We did not substitute any data for these missing temperature records.
SC (primary)	SC8DAI1	NWS ID 470703; RAWS; Minong, WI	46.13583 −91.98083	None.
SC (primary)	SC10DB1 SC10DD1	NOAA ID USC00478027; GHCND; Spooner, WI	45.8236 −91.8761	Tmin missing for 17 March 2009; Tmin and Tmax missing for all of July 2009; P missing for 23–25 January 2010. We substituted with data from the nearby station in Siren, WI (station KRZN), for July 2009. We did not fill the winter data gap in January 2010.
SC (mitigation)	SC10DB1 SC10DD1	WS ID KRZN; AWOS; Siren, WI	45.82346 −92.37369	
SC (primary)	SC12DA4 SC12DAI1	NWS ID 470804; RAWS; Hayward, WI	46.03111 −91.44900	P missing for 20–21 February 2012; Tmin and Tmax missing for 20 February–4 March 2012 and 30 March–1 April 2012 and 8–9 April 2012 and 11 April 2012 and 15 April 2012; Tmin, Tmax, and P missing for 1–6 January 2011. We substituted with data from Hayward, WI (station KHYR), for all data gaps.
SC (mitigation)	SC12DA4 SC12DAI1	WS ID KHYR; ASOS; Hayward, WI	46.0303 −91.4426	
NTL (primary)	TRL1DA1 TRL1DB1 TRL2DA1 TRL2DB1 TRL3DA1 TRL3DB1 TRL3DC1	NWS ID 471002; RAWS; Woodruff, WI	45.88972 −89.65222	P missing from 12–14 May 2011; Tmin and Tmax missing from 11–15 May 2011; P, Tmin, and Tmax missing for 28–29 March 2011. We substituted with data averaged from Arbor Vitae, WI (station KARV), and Minocqua, WI (GHCND:USC00475516), for all data gaps as Woodruff is midway between these two stations.
NTL (mitigation)	TRL1DA1 TRL1DB1 TRL2DA1 TRL2DB1 TRL3DA1 TRL3DB1 TRL3DC1	WS ID KARV; AWOS; Arbor Vitae, WI	45.9264 −89.7307	
NTL (mitigation)	TRL1DA1 TRL1DB1 TRL2DA1 TRL2DB1 TRL3DA1 TRL3DB1 TRL3DC1	NOAA ID USC00475516; GHCND; Minocqua, WI	45.8863 −89.7322	
NTL (primary)	TRL4DA1 TRL4DB1 TRL4DC1	NWS ID 470302; RAWS; Glidden, WI	46.14000 −90.00000	None.
UMR (primary)	PSP1 UMRP7	NOAA ID USC00478589; GHCND; Trempealeau, WI	43.9994 −91.4378	
UMR (primary)	TrNWRDA1	NOAA ID USC00472165; GHCND; Dodge, WI	44.1330 −91.5511	P, Tmin, and Tmax missing for 31 May 2011, but we did not substitute data for this date. Tmin for 16 January 2009 (−37.8 °C) was noticeably lower than for all other records during 2008–2012; we substituted the Tmin (31.1 °C) recorded at the two nearest stations.
UMR (primary)	UMRP4	NOAA ID USC00470124; GHCND; Alma, WI	44.32722 −91.91944	Tmin and Tmax missing for 16 March 2012 and 31 May 2012; Tmax missing for19 July 2012. We did substitute data for missing temperature records because they were sufficiently isolated in time to have little effect on our analyses.
UMR (primary)	UMRP10	NOAA ID USC00476827; GHCND; Prairie du Chien, WI	43.05150 −91.13490	Tmin missing for 2 January 2010. We did not substitute data for missing temperature record because it was sufficiently isolated in time to have little effect on our analyses

^1^ North American Datum 1983. ^2^ Data for these stations were from http://www.raws.dri.edu/index.html. ^3^ Data for these stations were from https://www.wunderground.com. ^4^ Data for these stations were from http://www.ncdc.noaa.gov/cdo-web/datasets.

## Appendix B

(Table A2) Cross reference for day-of-year, week number, and month starting date (the latter is for non-leap years); (Table A3) Cross reference of rolling seven-day satellite sensor products with weekly time steps; (Table A4) Cross reference of eight-day satellite sensor products with weekly time steps.

**Table A2:** Cross reference for day-of-year, week number, and month starting date (the latter is for non-leap years, 2009, 2010, and 2011). Starting dates in the column containing “Day-of-Year When Month Begins” are shifted by 1 following 28 February for leap years (2008 and 2012).

Ending Day-of-Year	Week Number	Day-of-Year When Month Begins	Ending Day-of-Year	Week Number	Day-of-Year When Month Begins
7	1	1 January (day #1)	189	27	
14	2		196	28	
21	3		203	29	
28	4		210	30	
35	5	1 February (day #32)	217	31	1 August (day #213)
42	6		224	32	
49	7		231	33	
56	8		238	34	
63	9	1 March (day #60)	245	35	1 September (day #244)
70	10		252	36	
77	11		259	37	
84	12		266	38	
91	13	1 April (day #91)	273	39	
98	14		280	40	1 October (day #274)
105	15		287	41	
112	16		294	42	
119	17		301	43	
126	18	1 May (day #121)	308	44	1 November (day #305)
133	19		315	45	
140	20		322	46	
147	21		329	47	
154	22	1 June (day #152)	336	48	1 December (day #335)
161	23		343	49	
168	24		350	50	
175	25		357	51	
182	26	1 July (day #182)	365	52	

**Table A3:** Cross reference of seven-day satellite sensor composite products with weekly time steps.

Day Number	Week Assigned	Day Number	Week Assigned	Day Number	Week Assigned	Day Number	Week Assigned	Day Number	Week Assigned	Day Number	Week Assigned
1–7	1	47–53	8	93–99	14	139–145	21	185–191	27	231–237	34
2–8	1	48–54	8	94–100	14	140–146	21	186–192	27	232–238	34
3–9	1	49–55	8	95–101	14	141–147	21	187–193	28	233–239	34
4–10	1	50–56	8	96–102	15	142–148	21	188–194	28	234–240	34
5–11	2	51–57	8	97–103	15	143–149	21	189–195	28	235–241	34
6–12	2	52–58	8	98–104	15	144–150	21	190–196	28		
7–13	2	53–59	8	99–105	15	145–151	22	191–197	28		
8–14	2	54–60	9	100–106	15	146–152	22	192–198	28		
9–15	2	55–61	9	101–107	15	147–153	22	193–199	28		
10–16	2	56–62	9	102–108	15	148–154	22	194–200	29		
11–17	2	57–63	9	103–109	16	149–155	22	195–201	29		
12–18	3	58–64	9	104–110	16	150–156	22	196–202	29		
13–19	3	59–65	9	105–111	16	151–157	22	197–203	29		
14–20	3	60–66	9	106–112	16	152–158	23	198–204	29		
15–21	3	61–67	10	107–113	16	153–159	23	199–205	29		
16–22	3	62–68	10	108–114	16	154–160	23	200–206	29		
17–23	3	63–69	10	109–115	16	155–161	23	201–207	30		
18–24	3	64–70	10	110–116	17	156–162	23	202–208	30		
19–25	4	65–71	10	111–117	17	157–163	23	203–209	30		
20–26	4	66–72	10	112–118	17	158–164	23	204–210	30		
21–27	4	67–73	10	113–119	17	159–165	24	205–211	30		
22–28	4	68–74	11	114–120	17	160–166	24	206–212	30		
23–29	4	69–75	11	115–121	17	161–167	24	207–213	30		
24–30	4	70–76	11	116–122	17	162–168	24	208–214	31		
25–31	4	71–77	11	117–123	18	163–169	24	209–215	31		
26–32	5	72–78	11	118–124	18	164–170	24	210–216	31		
27–33	5	73–79	11	119–125	18	165–171	24	211–217	31		
28–34	5	74–80	11	120–126	18	166–172	25	212–218	31		
29–35	5	75–81	12	121–127	18	167–173	25	213–219	31		
30–36	5	76–82	12	122–128	18	168–174	25	214–220	31		
31–37	5	77–83	12	123–129	18	169–175	25	215–221	32		
32–38	5	78–84	12	124–130	19	170–176	25	216–222	32		
33–39	6	79–85	12	125–131	19	171–177	25	217–223	32		
34–40	6	80–86	12	126–132	19	172–178	25	218–224	32		
35–41	6	81–87	12	127–133	19	173–179	26	219–225	32		
36–42	6	82–88	13	128–134	19	174–180	26	220–226	32		
37–43	6	83–89	13	129–135	19	175–181	26	221–227	32		
38–44	6	84–90	13	130–136	19	176–182	26	222–228	33		
39–45	6	85–91	13	131–137	20	177–183	26	223–229	33		
40–46	7	86–92	13	132–138	20	178–184	26	224–230	33		
41–47	7	87–93	13	133–139	20	179–185	26	225–231	33		
42–48	7	88–94	13	134–140	20	180–186	27	226–232	33		
43–49	7	89–95	14	135–141	20	181–187	27	227–233	33		
44–50	7	90–96	14	136–142	20	182–188	27	228–234	33		
45–51	7	91–97	14	137–143	20	183–189	27	229–235	34		
46–52	7	92–98	14	138–144	21	184–190	27	230–236	34		

**Table A4:** Cross reference of eight-day satellite sensor composite products with weekly time steps.

8-Day Interval	Days of Year	Week Assigned ^1^
1	1–8	1
2	9–16	2
3	17–24	3
4	25–32	4
5	33–40	6
6	41–48	7
7	49–56	8
8	57–64	9
9	65–72	10
10	73–80	11
11	81–88	12
12	89–96	14
13	97–104	15
14	105–112	16
15	113–120	17
16	121–128	18
17	129–136	19
18	137–144	20
19	145–152	22
20	153–160	23
21	161–168	24
22	169–176	25
23	177–184	26
24	185–192	27
25	193–200	28
26	201–208	30
27	209–216	31
28	217–224	32
29	225–232	33
30	233–240	34

^1^ There were no eight-day intervals assigned to weeks 5, 13, 21, and 29 because there are four fewer eight-day intervals than weeks in the January–August time frame.
