# Supplementary material for: Challenges in Complementing Data from Ground-Based Sensors with Satellite-Derived Products to Measure Ecological Changes in Relation to Climate—Lessons from Temperate Wetland-Upland Landscapes
Source: Sensors (Basel). 2018 Mar 16;18(3):880. doi: 10.3390/s18030880 (PMC5876606; doi:10.3390/s18030880)
Supplement: Supplementary file 1 [file sensors-18-00880-s001.pdf]

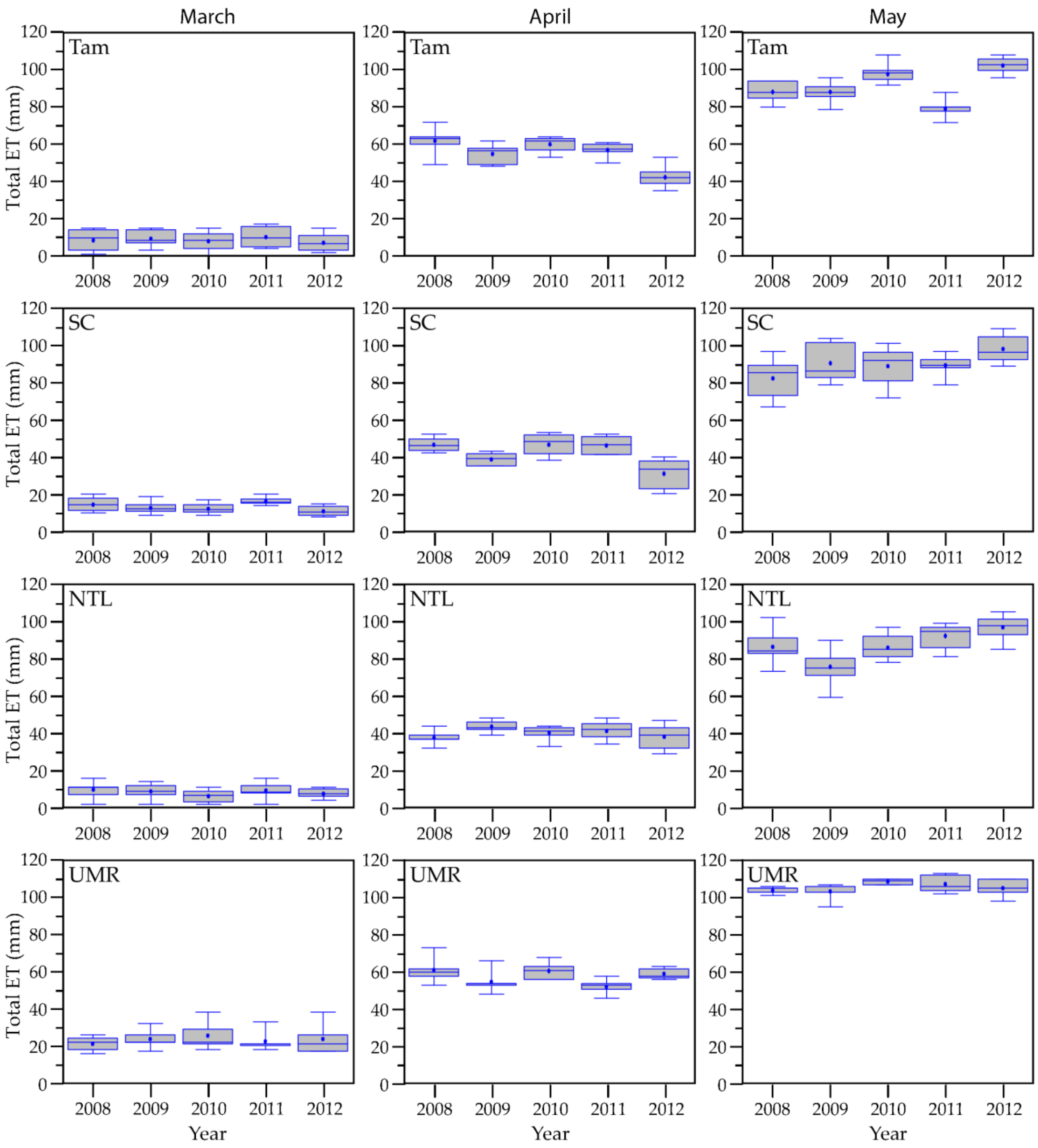

Figure S1. Interannual differences in early season monthly evapotranspiration (ET). Tam=Tamarac National Wildlife Refuge. SC=St. Croix National Scenic Riverway. NTL=North Temperate Lakes Long-term Research Area. UMR=Upper Mississippi River floodplain.

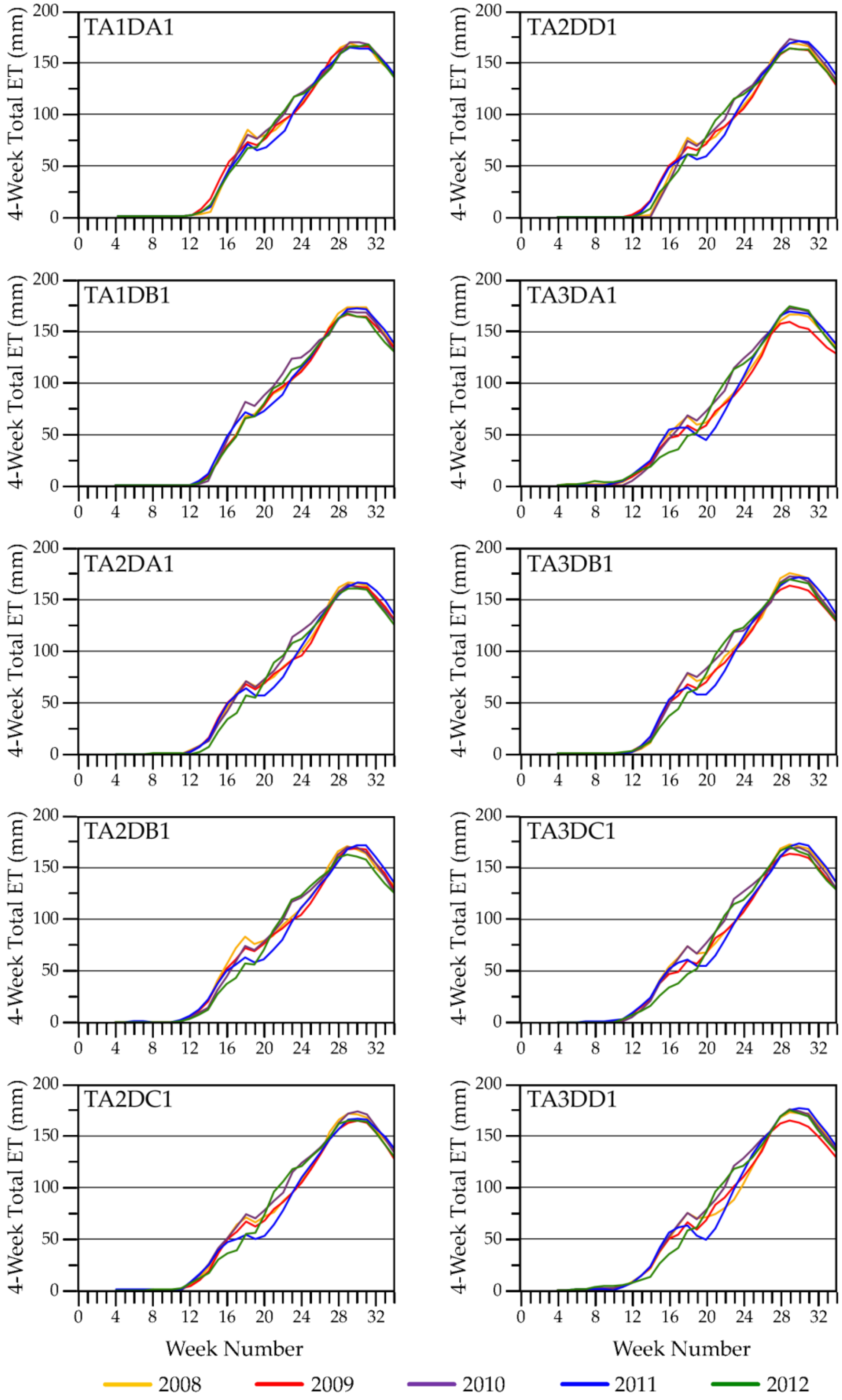

Figure S2a. Four-week total evapotranspiration (ET), incremented weekly, for blocks in the Tamarac National Wildlife Refuge study area.

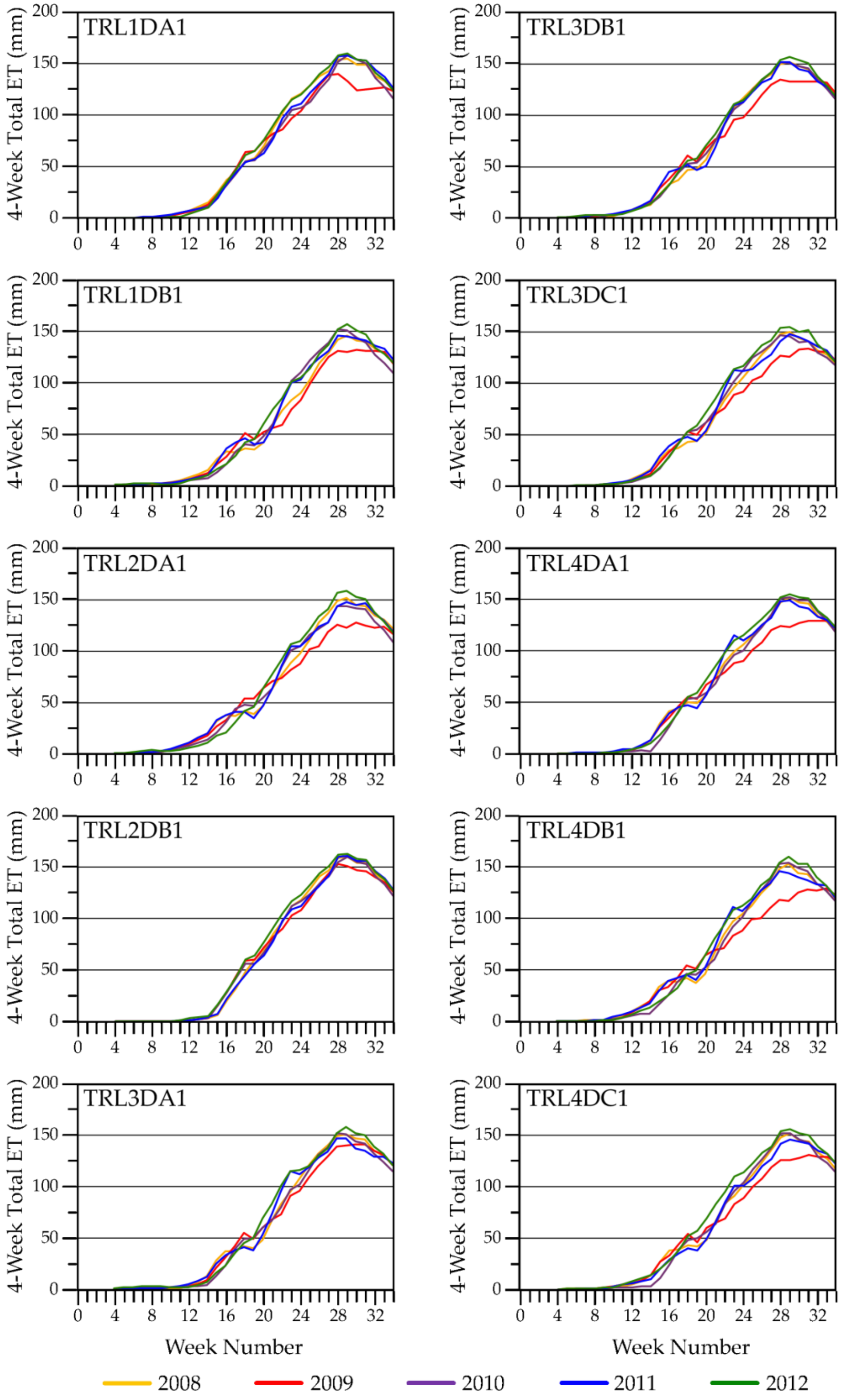

Figure S2b. Four-week total evapotranspiration (ET), incremented weekly, for blocks in the North Temperate Lakes Long-term Research Area study area.

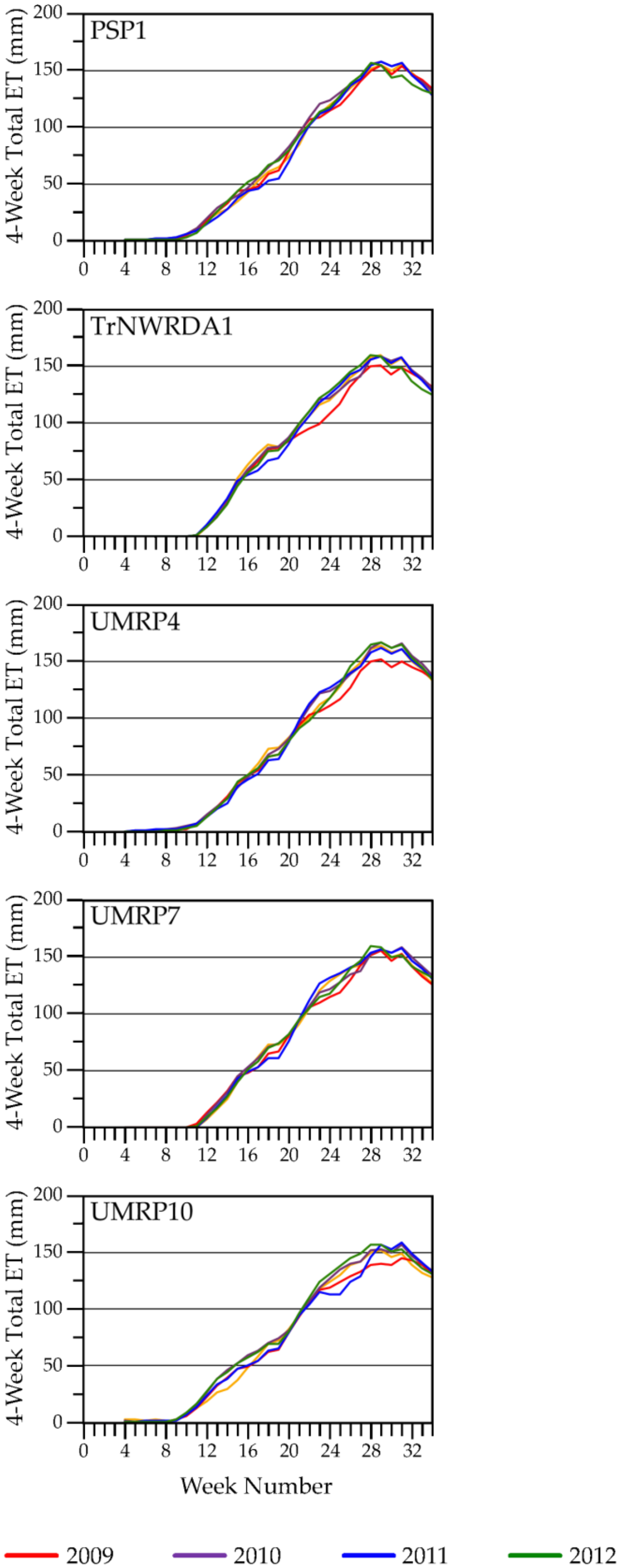

Figure S2c. Four-week total evapotranspiration (ET), incremented weekly, for blocks in the Upper Mississippi River floodplain study area.

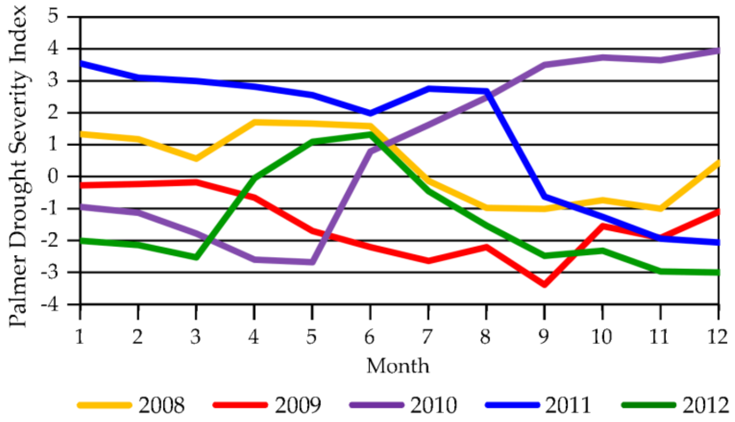

Figure S3. Palmer Drought Severity Index (PDSI [65]) for the NOAA climate division (WICDO1) representing the St. Croix National Scenic Riverway study area (data obtained from the National Climate Data Center[66]). Negative values of the PDSI denote dry conditions and positive values denote wet conditions. Values 0 to −0.5 = normal conditions; −0.5 to −1.0 = incipient drought; −1.0 to −2.0 = mild drought; −2.0 to −3.0 = moderate drought; −3.0 to −4.0 = severe drought; and greater than −4.0 = extreme drought. Similar adjectives are used for positive values of wet spells.

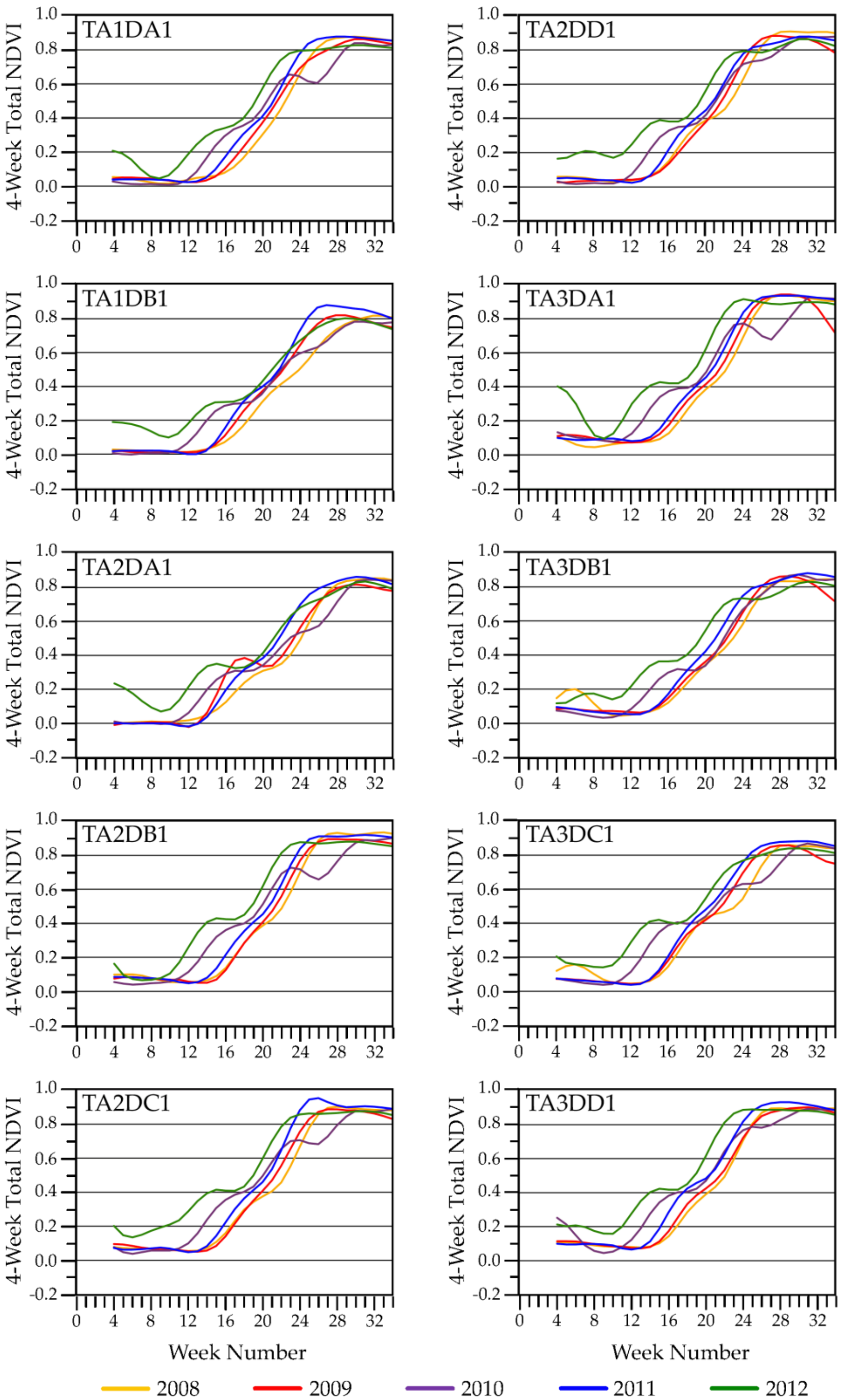

Figure S4a. Four-week total Normalized Difference Vegetation Index (NDVI), incremented weekly, for blocks in the Tamarac National Wildlife Refuge study area.

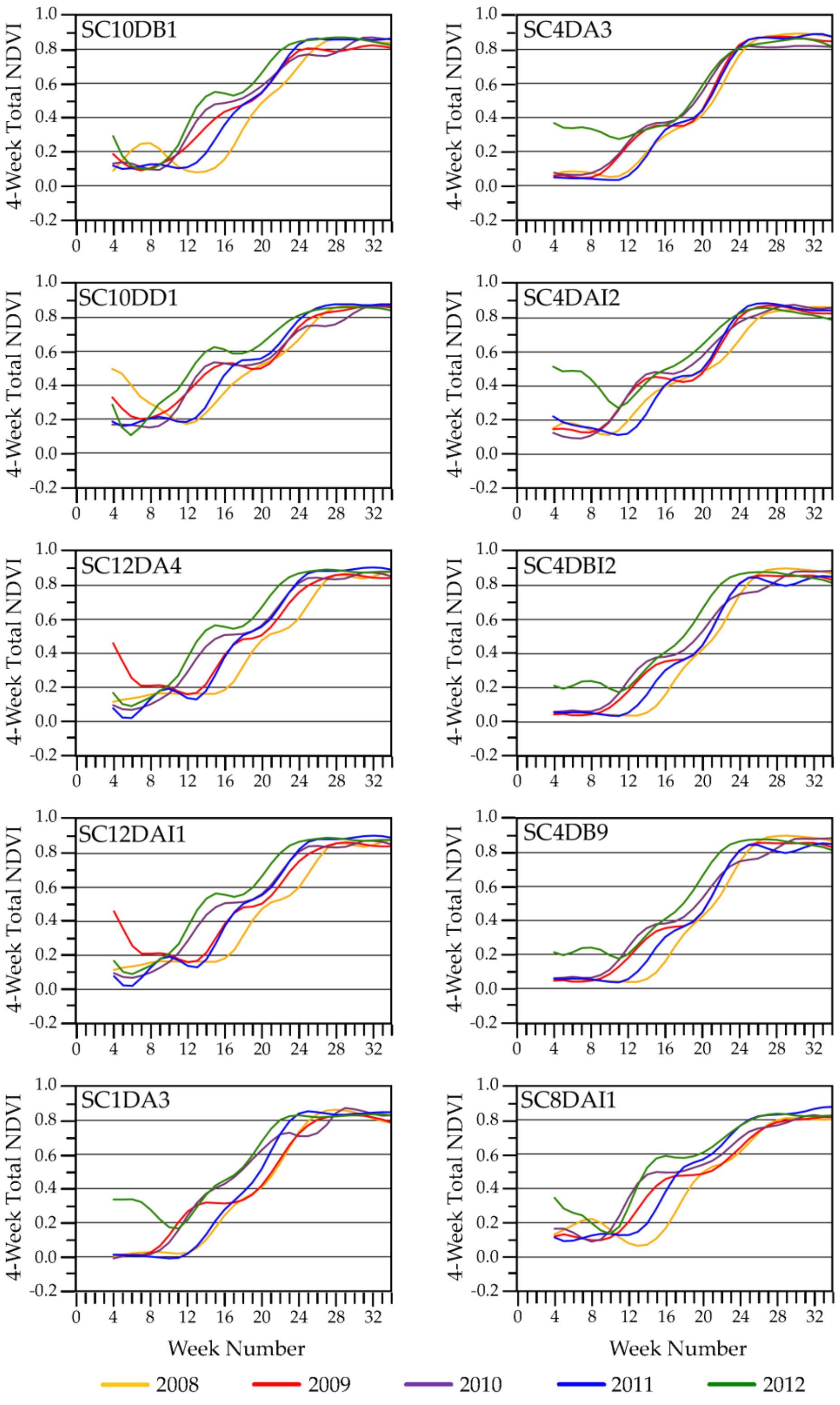

Figure S4b. Four-week total Normalized Difference Vegetation Index (NDVI), incremented weekly, for blocks in the St. Croix National Scenic Riverway study area.

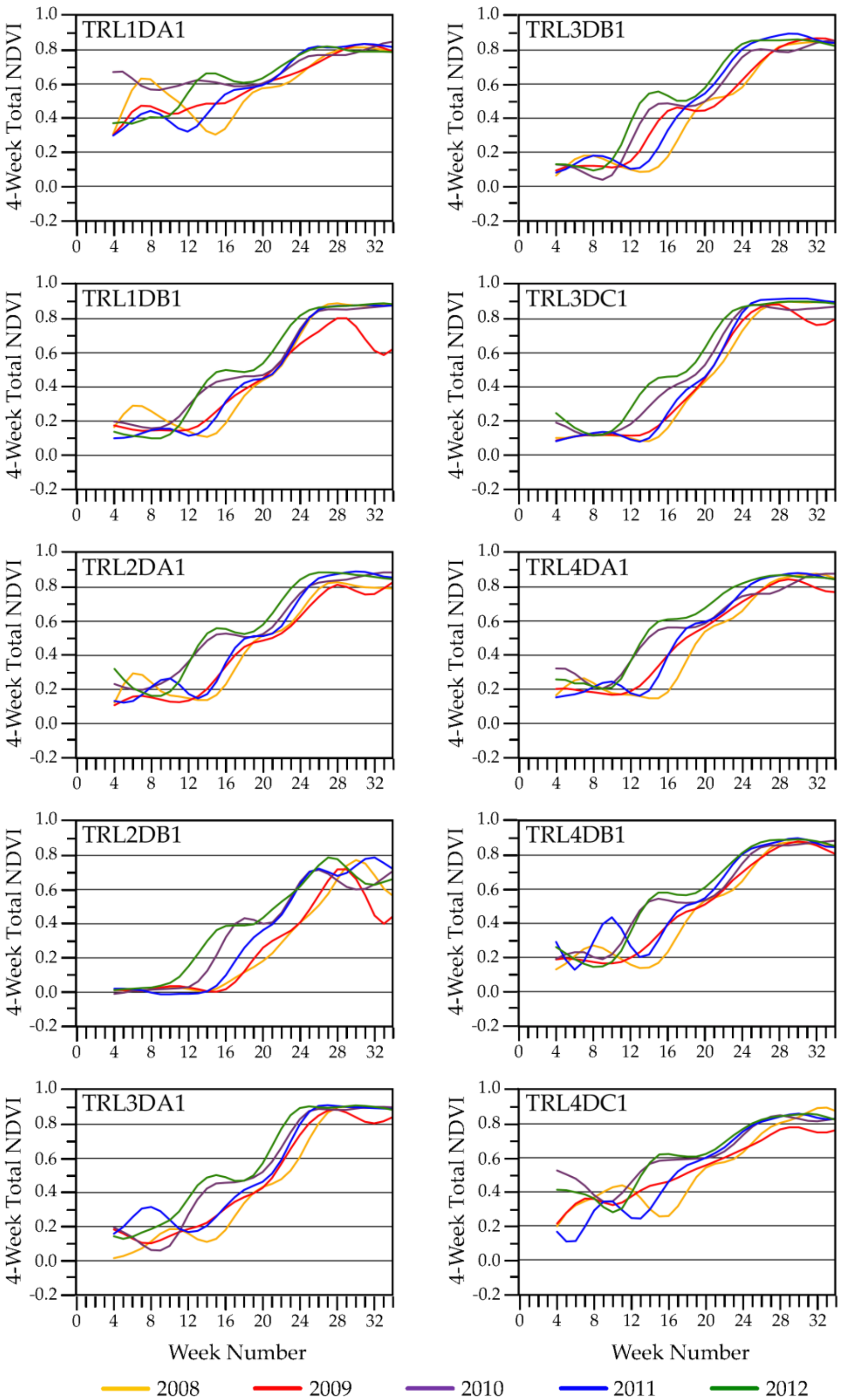

Figure S4c. Four-week total Normalized Difference Vegetation Index (NDVI), incremented weekly, for blocks in the North Temperate Lakes Long-term Research Area study area.

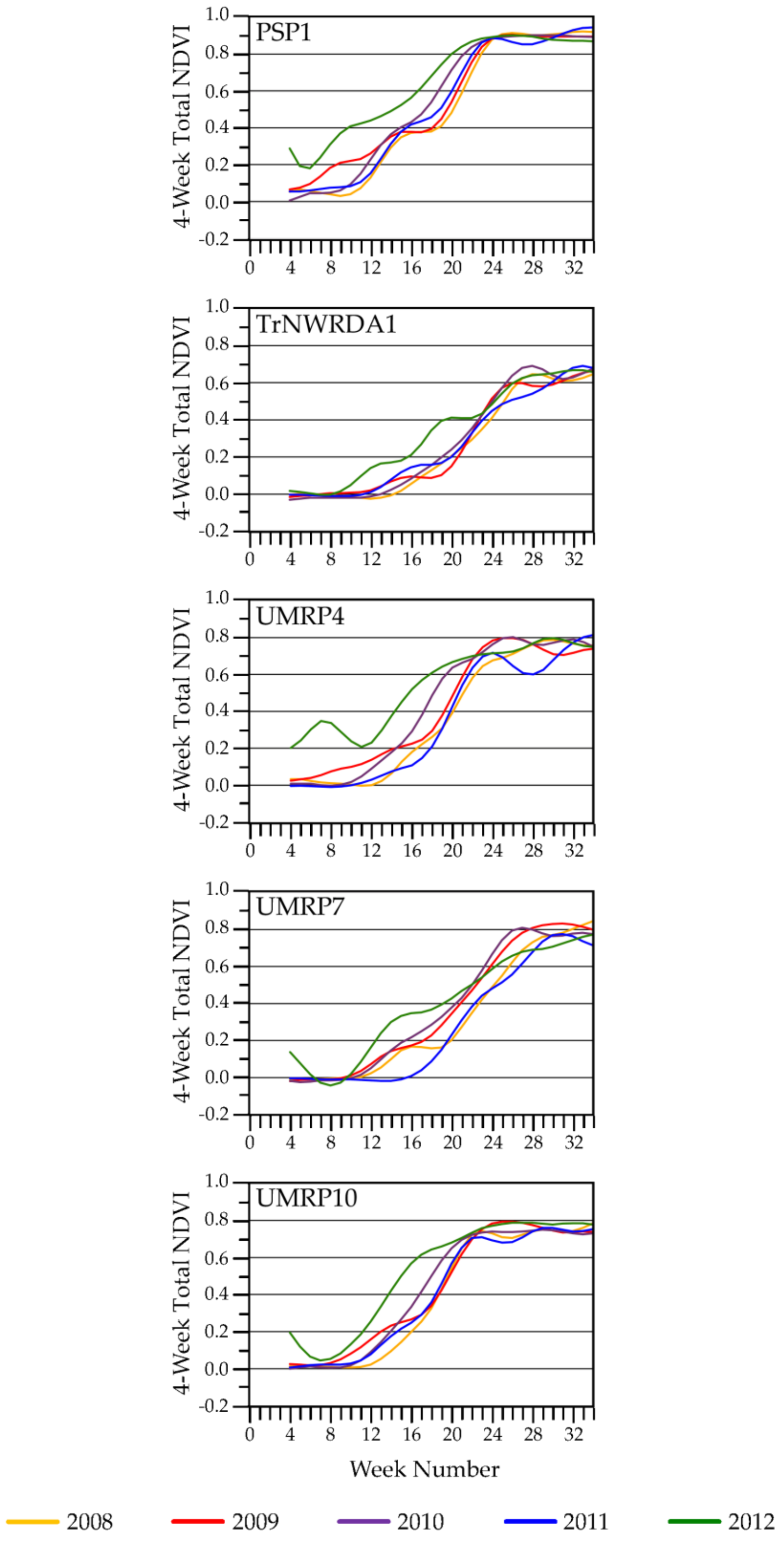

Figure S4d. Four-week total Normalized Difference Vegetation Index (NDVI), incremented weekly, for blocks in the Upper Mississippi River floodplain study area.

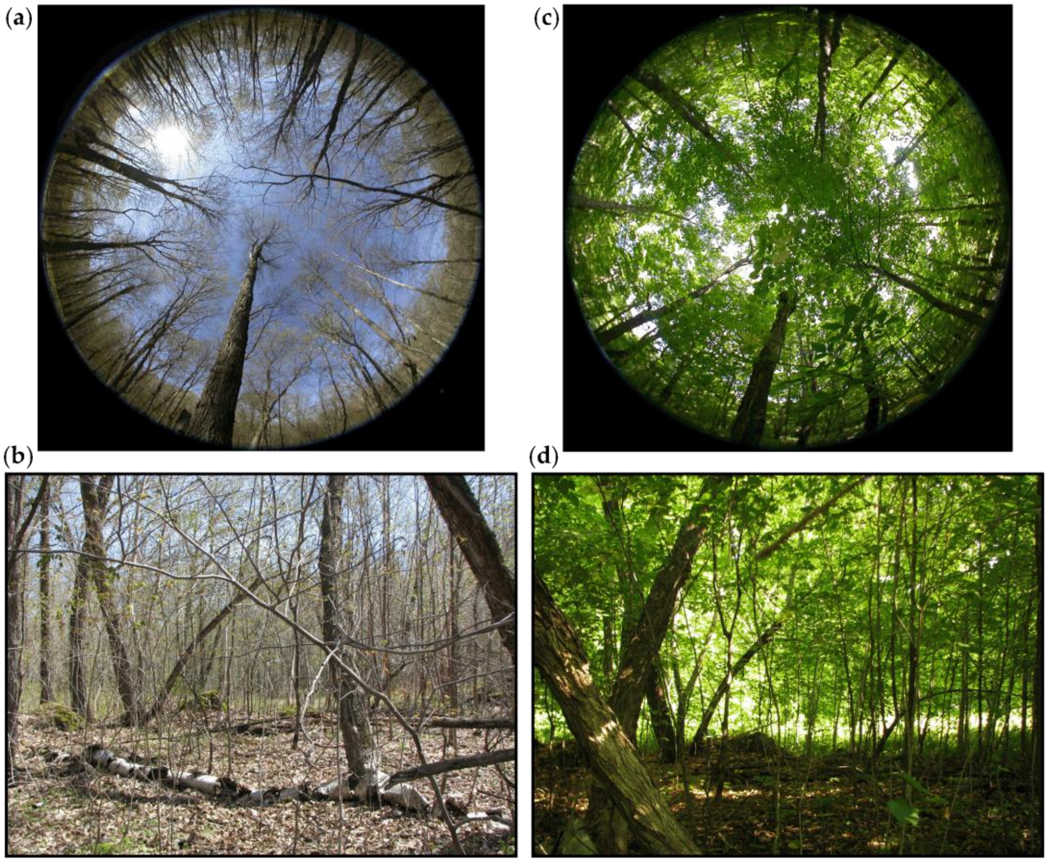

Figure S5. Location near field site TA1DB1 in the Tamarac National Wildlife Refuge study area showing early stages of leaf-out in the canopy (**a**) and on the ground (**b**) on 17 May 2009, compared with full leaf-out conditions on 7 September 2009 in the canopy (**c**) and on the ground (**d**). Note, we took the photos in May and September from the same location, but not from the same exact position.

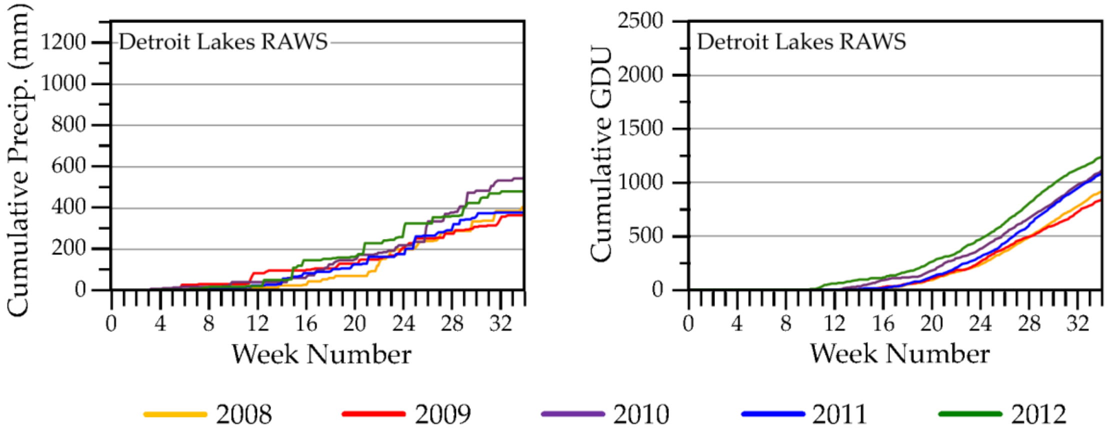

Figure S6a. Cumulative precipitation and growing degree units (GDU) from weather stations we used to represent the Tamarac National Wildlife Refuge study area from January through August for 2008–2012. See Appendix A for descriptions of weather stations and list of associated study sites.

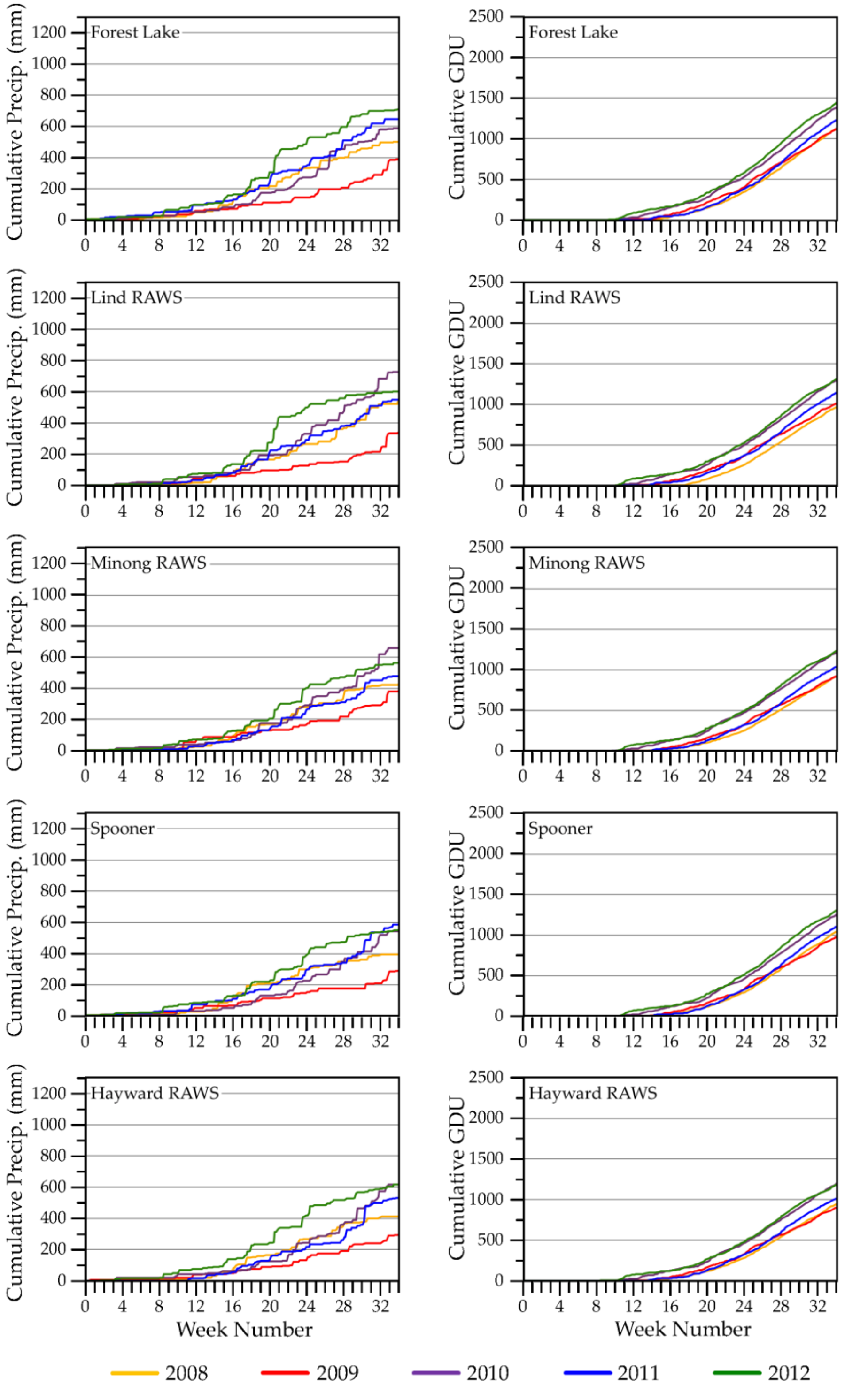

Figure S6b. Cumulative precipitation and growing degree units (GDU) from weather stations we used to represent the St. Croix National Scenic Riverway study area from January through August for 2008–2012. See Appendix A for descriptions of weather stations and list of associated study sites.

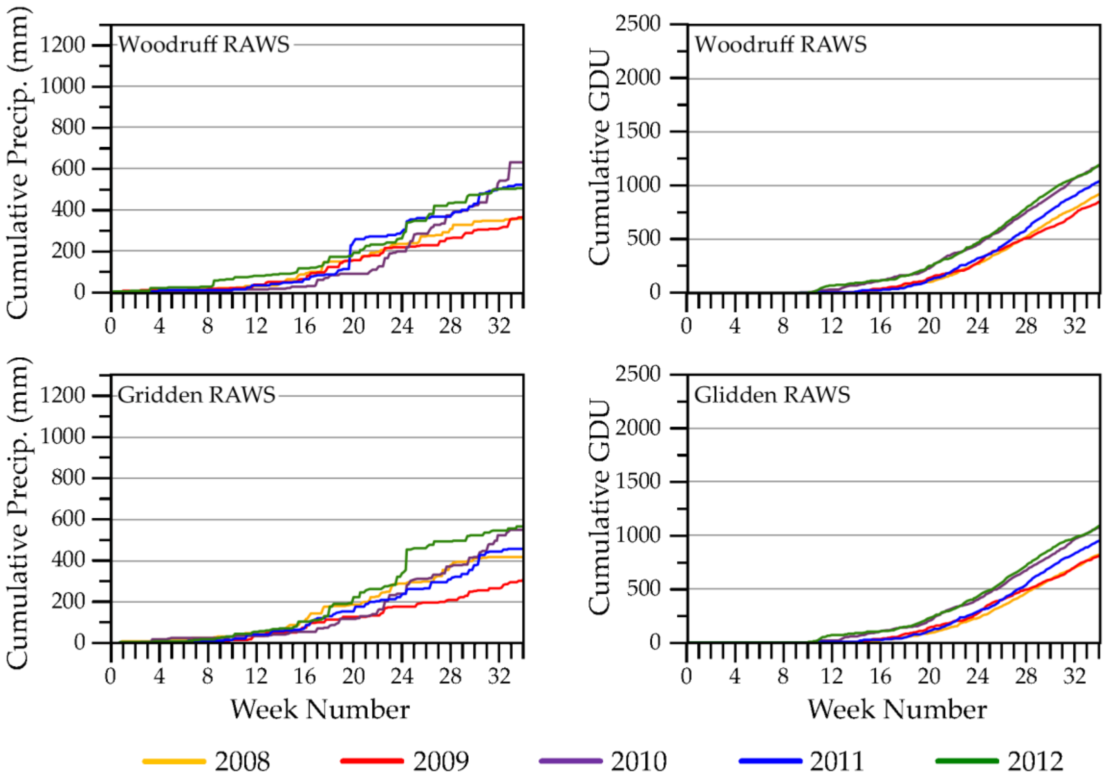

Figure S6c. Cumulative precipitation and growing degree units (GDU) from weather stations we used to represent the North Temperate Lakes Long-term Research Area study area from January through August for 2008–2012. See Appendix A for descriptions of weather stations and list of associated study sites.

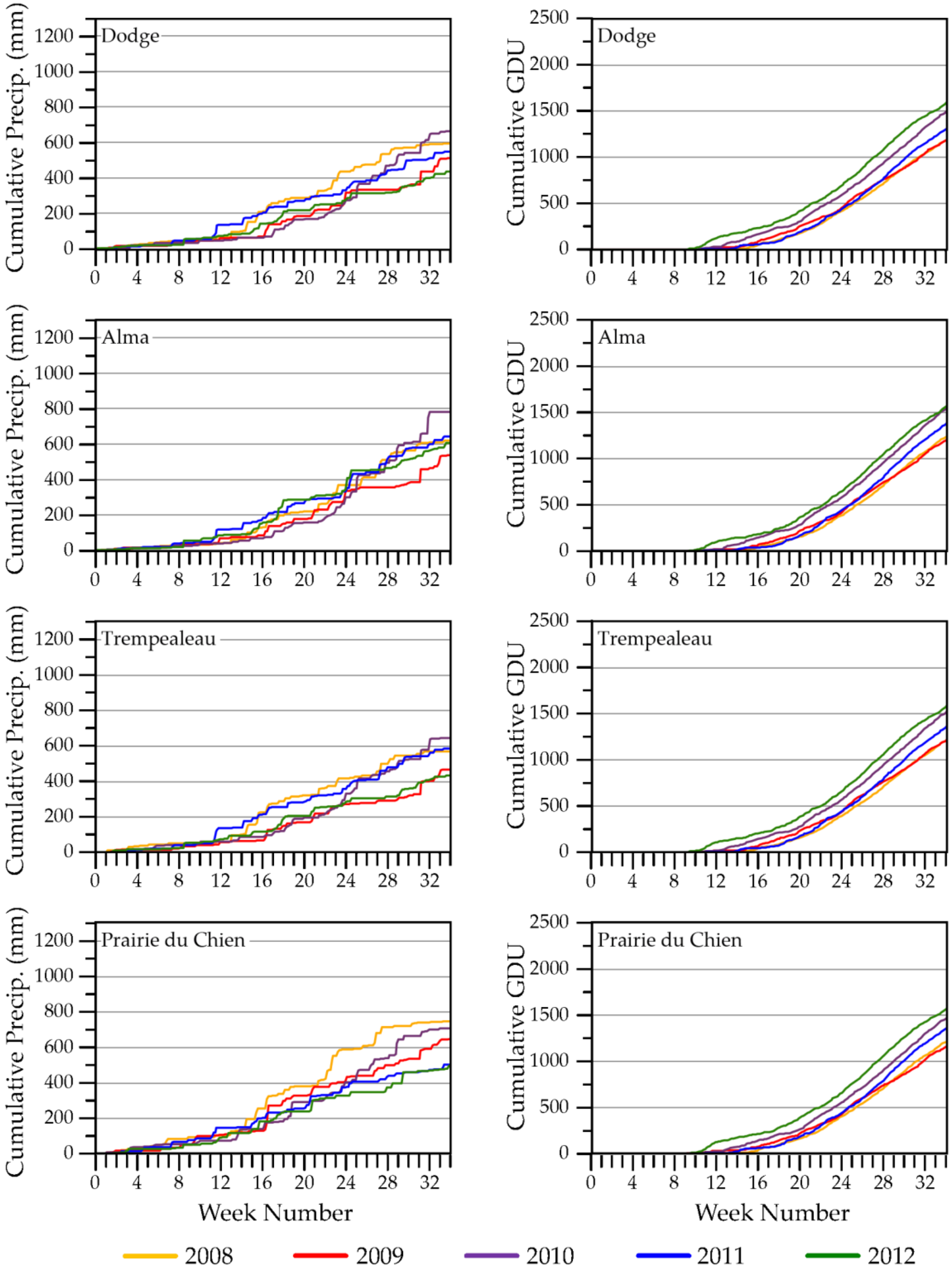

Figure S6d. Cumulative precipitation and growing degree units (GDU) from weather stations we used to represent for the Upper Mississippi River floodplain study area from January through August for 2008–2012. See Appendix A for descriptions of weather stations and list of associated study sites.
